# Robotic versus laparoscopic hepatectomy for liver malignancies (ROC'N'ROLL): a single-centre, randomised, controlled, single-blinded clinical trial

**DOI:** 10.1016/j.lanepe.2024.100972

**Published:** 2024-06-24

**Authors:** Emrullah Birgin, Marie Heibel, Svetlana Hetjens, Erik Rasbach, Christoph Reissfelder, Patrick Téoule, Nuh N. Rahbari

**Affiliations:** a Department of General and Visceral Surgery, Ulm University Hospital, Ulm, Germany; b Department of Surgery, Universitätsmedizin Mannheim, Medical Faculty Mannheim, Heidelberg University, Mannheim, Germany; c Department of Medical Statistics and Biomathematics, Medical Faculty Mannheim, Heidelberg University, Mannheim, Germany

**Keywords:** Liver resection, Minimally invasive hepatectomy, DaVinci®, Robot-assisted, Randomized controlled trial

## Abstract

**Background:**

Robotic hepatectomy (RH) has been increasingly adopted for the treatment of liver malignancies despite lacking evidence from randomised trials. We aimed to determine the effect of RH compared to laparoscopic hepatectomy (LH) on quality of life in patients undergoing minimally invasive hepatectomy for liver malignancies.

**Methods:**

This single-blinded, randomised trial was conducted at a tertiary care academic centre (DRKS00027531). Patients with resectable liver malignancies were assessed for eligibility and randomly assigned to either RH or LH with stratification by type of malignancy and difficulty of resection. Patients were blinded to the treatment allocation. The primary outcome was the mean quality of life within 90 days after surgery, measured with the role functioning scale of the European Organisation for Research and Treatment of Cancer QLQ-C30 questionnaire. Secondary outcomes included operating time, morbidity, blood loss, conversion rate, postoperative recovery, and resection margin status.

**Findings:**

Between February 21, 2022, and Sep 18, 2023, 80 patients (RH: n = 41, LH: n = 39) were included and analysed on an intention-to-treat basis. Role functioning scores did not differ between RH and LH (mean [SD], 74.3 [23.3] versus 79.6 [22.3]; mean difference −5.3, 95% CI −15.6 to 5.1, p = 0.547). The comprehensive complication index was not significantly different between the study groups (8.9 [23.1] versus 15.5 [23.9], p = 0.137). There were no differences in other perioperative outcomes.

**Interpretation:**

RH yielded similar outcomes in quality of life and can be considered a safe alternative to LH.

**Funding:**

None.

**Research in context**

**Evidence before this study**

We performed a systematic literature review using PubMed on search terms including “robotic hepatectomy”, “laparoscopic hepatectomy” and “liver malignancies” to find studies comparing robotic and laparoscopic hepatectomy for liver malignancies prior to the preparation and start of our trial (as of December 2021). No language restriction was applied. A total of 238 results were obtained and screened for original studies. We identified 11 retrospective cohort studies comparing robotic and laparoscopic hepatectomies in liver malignancies, while none of the studies assessed quality of life between both approaches. One randomised trial assessed quality of life in minimally invasive hepatectomy as a secondary endpoint and found it to be improved after laparoscopic hepatectomy compared to open hepatectomy. Three meta-analyses were published with inconsistent findings in terms of morbidity, blood loss, conversion rates, and postoperative stay including primarily retrospective studies. No randomised trials were identified. Hence, a low level of evidence with high risk of selection and attrition bias was found in literature.

**Added value of this study**

This is the first randomised trial to compare robotic hepatectomy and laparoscopic hepatectomy in patients with liver malignancies. We found no significant differences in patient reported outcomes and negative resection margins between the study groups. Perioperative morbidity in line with the comprehensive complication index was comparable between the groups.

**Implications of all the available evidence**

Robotic hepatectomy for liver malignancies represent a safe alternative to laparoscopic hepatectomy. We found no changes of quality of life, oncological outcomes, and morbidity between both surgical modalities.

## Introduction

Laparoscopic hepatectomy (LH) has proved efficacious in the treatment of liver malignancies, with no disadvantage in oncological outcomes.^1^, ^2^, ^3^ Two randomised trials showed superiority of LH over open hepatectomy in patients with colorectal liver metastasis regarding postoperative complications and hospital stay.^1^^,^^3^ Another randomised trial demonstrated faster recovery following LH in cases of primary liver cancer.^2^

Following oncological surgery, patients' functional roles in daily life remain a pivotal health value and a significant oncological health outcome, alongside objective measures such as negative resection margins or postoperative morbidity.^4^^,^^5^ Moreover, role functioning represents a multi-dimensional, composite assessment of patients' well-being, which reflects surgical outcomes, oncological results, and postoperative recovery. Hence, role functioning covers all aspects of surgical and oncological outcomes, including perioperative morbidity, recovery, and psychological well-being. In doing so, trials based on patient-reported outcome measures demonstrate tolerability of surgical intervention from patients’ perspective and might change clinical guidelines.^6^ One randomised controlled trial reported higher quality of life (QoL) after LH compared to open hepatectomy.^7^

However, the contemporary landscape of minimally invasive surgery is undergoing a paradigm shift from laparoscopic approaches towards robotic surgery despite lack of level one evidence.^8^^,^^9^ The widespread utilization of the robotic platform is mainly attributed to its visualization, surgical precision, increasing institutional availability, and dexterity in minimally invasive liver surgery.^10^^,^^11^ A recent meta-analysis supported the advantages of robotic hepatectomy (RH) in terms of reduced morbidity, blood loss, conversion rates, and postoperative stay compared to LH.^12^ However, several other meta-analyses yielded inconclusive findings concerning postoperative outcomes when comparing these two minimally invasive modalities.^13^, ^14^, ^15^, ^16^ To date, no randomised controlled trial has been reported comparing RH to conventional LH. Consequently, the available evidence is solely derived from uncontrolled cohort studies.^17^ Nevertheless, RH holds promise in reducing morbidity, blood loss, and the need for conversion to open surgery, owing to technological advancements in minimally invasive techniques, thereby resulting in enhanced QoL and accelerated recovery.^18^ Given the limited evidence on short-term oncological and patient-reported outcomes (PRO) after RH, we conducted the first randomised clinical trial to test the hypothesis that RH improves QoL compared to LH in patients undergoing liver resection for malignancies.

## Methods

### Study design and participants

This study was an investigator-initiated, prospective, single-centre, randomised, single-blinded, parallel-group clinical trial comparing RH to LH for patients with liver malignancies. All surgeries were performed at the Department of Surgery, University Hospital Mannheim, Heidelberg University. Eligible patients were aged 18 years or older undergoing curative-intent minimally invasive (robotic or laparoscopic) hepatectomy for confirmed or suspected primary and secondary liver malignancies after multidisciplinary team review. Hepatobiliary surgeons confirmed resectability to achieve R0 resection before surgery. Patients requiring hepatectomy with vascular reconstruction and/or extrahepatic resections were excluded. Further exclusion criteria included patients with American Society of Anesthesiologists (ASA) level 4 or higher, impaired mental state or inability to complete questionnaires. Preoperative liver function was assessed by liver volumetry (in case of major hepatectomy), while laboratory tests including prothrombin time, platelets, liver enzymes, and evaluation of the Child-Pugh Score was used routinely in all patients. Enrolment took place from February 21, 2022, to September 18, 2023, with a follow-up of 90 days (last visit on December 18, 2023). The study was approved by the institutional review board of Heidelberg University (2021-672). The trial was registered with the German Clinical Trial Register (DRKS00027531) before enrolment. The trial protocol is available in Supplement 1. All participants provided written informed consent. Patients received no financial compensation. There was no funding source for this study.

### Randomization and blinding

Participants were randomised on a 1:1 ratio and stratified by type of malignancy (primary versus secondary liver malignancy) and difficulty of resection as determined by the Iwate scoring system (difficulty score 1–5 versus 6–12, based on tumor location, tumor size, proximity to major vessels, liver function, and hand-assisted hybrid procedures) at screening.^19^ Randomisation was performed within the Research Electronic Data Capture (REDCap) system using a random number of blocks with block sizes of two and four to receive either RH or LH. The randomisation was performed by trial staff who were not involved in the patient treatment. Participants were told at randomisation that a minimally invasive hepatectomy will be performed, while the specific type of approach was kept secretly until the end of study (unblinding was made at the last follow-up visit). In the medical charts' (e.g., operation note, discharge note) there was no report of the specific surgical approach “robotic or laparoscopic hepatectomy” and the term was masked by “minimally invasive hepatectomy”. Anesthesia was performed in the induction rooms outside the operating theatres. After surgery, no specific dressings were used as both groups underwent minimally invasive approaches. The blinding was monitored by the clinical trial staff. In addition, outcomes were assessed and analysed by blinded observers, whereas surgeons were not blinded to the intervention (single-blinded trial).^20^

### Interventions

All surgeries were performed by highly experienced minimally invasive hepatobiliary surgeons. Each trial surgeon had to fulfill two criteria to be eligible: 1. at least 50 minimally invasive hepatectomies per year by laparoscopic or robotic approach; 2. minimum of 25 robotic hepatectomies in total. Of note, each trial surgeon had conducted a minimum of 150 laparoscopic hepatectomies before participating in the trial while a case-splitting of hepatectomies was allowed if two primary surgeons performed distinct liver resections during the same operative session (i.e., partial hepatectomies, segmentectomies). Both robotic and laparoscopic hepatectomies were performed according to a standardized technique as described previously.^21^, ^22^, ^23^ Patients were placed in a reverse Trendelenburg position. Pneumoperitoneum was established at 12 mmHg and an intraoperative ultrasound was performed to determine the transection plane and confirm resectability. During parenchymal transection, the pneumoperitoneum was raised to 15–18 mmHg. Intermittent Pringle maneuver was performed using an umbilical tape or Foley catheter around the hepatoduodenal ligament. In the LH group, hepatic transection was performed using bipolar forceps and a crush-clamping technique in combination with sealing devices (LigaSure™, Medtronic, Minneapolis, MN, USA; Thunderbeat™, Olympus Medical Systems Corp., Tokyo, Japan), while a scissor hepatectomy technique or a vessel sealer was applied using the daVinci *Xi* or *X* platform (Intuitive Surgical, Sunnyvale, CA, USA) in the RH group.^24^ In both groups, intrahepatic vessels were divided using linear staplers, Hem-o-lok clips, or titanium clips. A routine lymphadenectomy was performed only in patients with suspected cholangiocarcinoma. The specimen was extracted using a Pfannenstiel incision or by reopening old abdominal scars. Intraabdominal drains were not placed routinely. Perioperative care was identical in both groups.

### Outcomes

The primary endpoint was the mean QoL measured with the role functioning scale of the European Organisation for Research and Treatment of Cancer QLQ-C30 questionnaire at 30, 60, and 90 days after surgery.^25^ Secondary endpoints included operating time, blood loss, conversion rate, postoperative adverse events, PRO measures (i.e., EuroQoL 5D-5L and visual analogue scale, QLQ-C30 outcomes), resection margin status, readmission rate and 90-day mortality. Specific posthepatectomy outcomes including posthepatectomy liver failure, posthepatectomy hemorrhage and posthepatectomy bile leakage were graded according to the International Study Group of Liver Surgery definitions.^9^^,^^10^^,^^26^ Postoperative morbidity was graded according to the Clavien Dindo classification with calculation of the comprehensive complication index.^27^ A detailed description of endpoints is available in Supplement 1**.**

### Statistical analysis

The sample size was based on the hypothesis of a higher QoL after RH within 90 days after surgery as compared to LH. A mean difference of 13% (standard deviation 20) in the role functioning scale was deemed clinically meaningful and used for power calculation in line with previous trials on quality of life after liver surgery.^7^^,^^28^, ^29^, ^30^, ^31^ The approach of sample size calculation based on clinically meaningful differences on the QoL scale was in line with other randomised trials using QoL as primary endpoint.^32^, ^33^, ^34^ A total of 76 patients (38 in each arm) provided 80% power (α = 0.05) to detect this difference with a two-sided significance. We anticipated an exclusion rate of 15% after randomization due to withdrawals, unresectability, or loss to follow-up, and thus anticipated enrolling 90 patients.

Missing data at random for PRO measures were imputed if at least one postoperative assessment was available. Multiple imputation was performed using the Markov Chain Monte Carlo method with 50 imputation datasets based on baseline demographic values.^35^ Patients who died in the postoperative period or were lost to follow-up without any postoperative PRO assessments were excluded from the primary endpoint analysis (complete case-only analysis) without data imputation.^36^ We further performed additional imputation analyses with imputing “0″ for all missing PRO values (equivalent to worst outcome).^36^, ^37^, ^38^ All outcomes were analysed on an intention-to-treat basis. Continuous data were described using mean or median values with standard deviation (SD) or interquartile range (IQR) depending on the pattern of distribution. Discrete data were described by frequencies and percentages. The changes of PRO measures from baseline were presented using mean differences with corresponding 95% confidence interval (CI), and odds ratios (OR) were calculated for binary variables. Comparisons between continuous data were performed with the Mann–Whitney U or independent samples t-test as appropriate. The Chi-square or Fisher's exact test was used to compare discrete data as appropriate. The primary endpoint was assessed with an analysis of covariance (ANCOVA), adjusting for age and the role functioning score before surgery. Other PRO measures were analysed accordingly using ANCOVA analysis. We performed additional sensitivity analyses to address potential imbalances of baseline characteristics on outcomes. Statistical analyses were performed in R, version 3.6.1 (R Foundation for Statistical Computing) and SAS 9.4 (SAS Institute Inc., Cary, NC) from December 20, 2023, to April 14, 2024.

## Results

### Patients

**Fig. 1:**
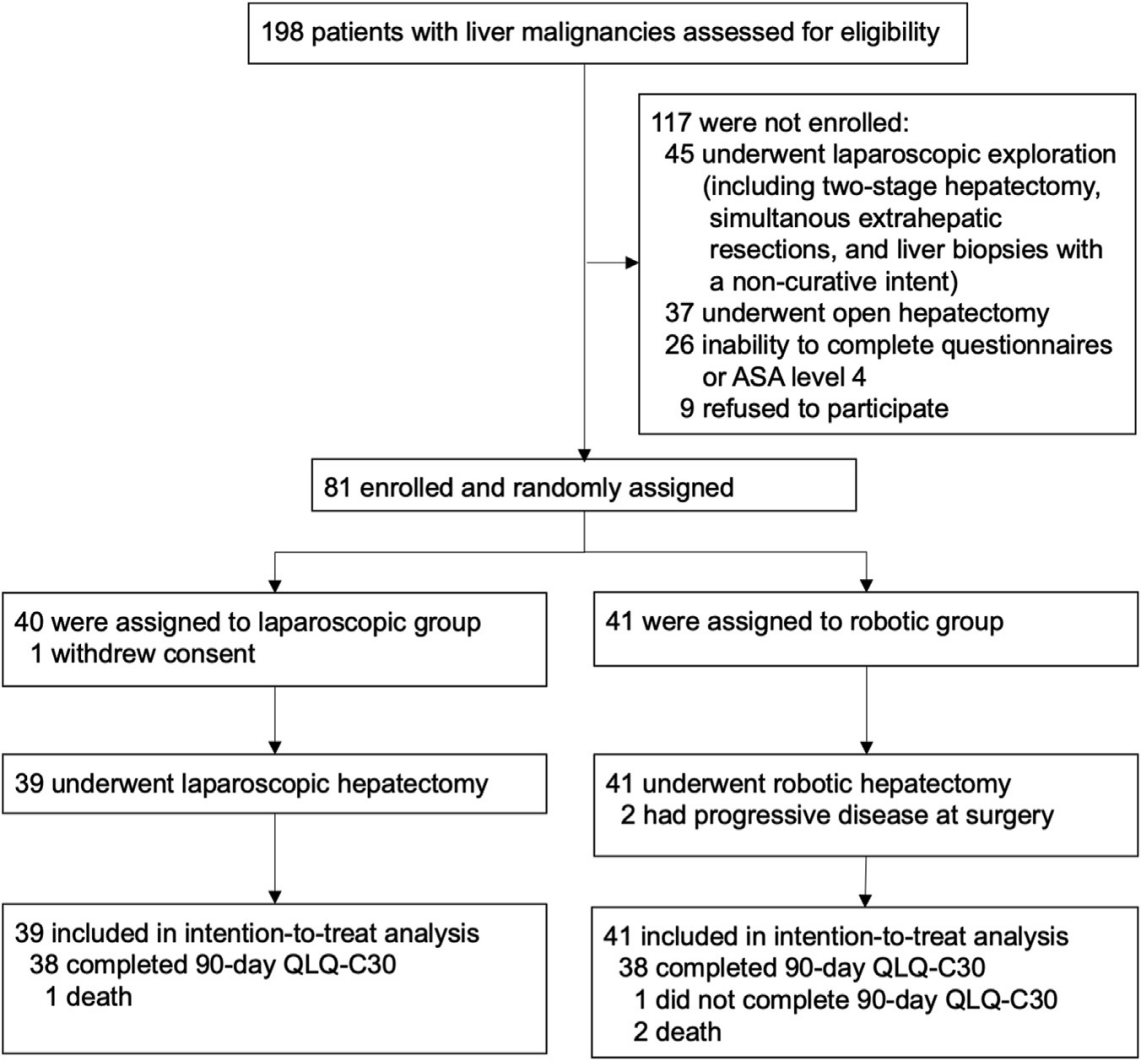
**Patient flow chart**.

A total of 81 patients were enrolled and randomly assigned to either the RH or LH group, as shown in Fig. 1. One patient in the LH group withdrew consent and was excluded, leaving 80 patients in the intention-to-treat analysis (41 in the RH and 39 in the LH group). All patients completed the trial. The median age was 66 [55–75] years, with 47 being men (59%). The majority underwent hepatectomy for secondary liver malignancies (n = 48, 60%) with a median Iwate difficulty score of six points [5–10]. Notably, 55 patients (69%) had previous abdominal surgeries, and of these patients 16 (20%) were included for repeat hepatectomies. A total of 21 patients (27%) had multifocal lesions. Patients’ clinicopathological characteristics are presented in Table 1. Age, BMI, and the comorbidity index were well balanced between the study groups. In the RH group, there were 20 (49%) male patients as compared to 27 (69%) in the LH group. A total of 21 (51%) patients had an ASA III status in the RH group compared to 14 (36%) in the LH group.

**Table 1:** Baseline demographics.

Characteristic	LH (n = 39)	RH (n = 41)
**Age, median (IQR), years**	65 (55–71)	66 (56–75)
**BMI, median (IQR), kg/m**^**2**^	27 (24–30)	26 (23–27)
**Sex ratio, Male:Female**	27:12	20:21
**Education status**		
1st–4th grades	1 (3)	1 (2)
5th–10th grades	19 (49)	22 (54)
11th–13th grades	9 (23)	10 (24)
University	10 (26)	8 (20)
**Employment status**		
Employed	16 (41)	11 (27)
Retired	22 (56)	27 (66)
Unemployed	1 (3)	3 (7)
**Residence area**		
Urban	3 (8)	9 (22)
Suburban	25 (64)	21 (51)
Rural	11 (28)	11 (27)
**Household income**		
<900 €	1 (3)	1 (2)
900 €—2000 €	8 (21)	8 (20)
2000 €—3600 €	14 (36)	9 (22)
3600 €—5000 €	2 (5)	4 (10)
>5000 €	6 (15)	6 (15)
Missing	8 (21)	13 (32)
**ASA status**		
I	2 (5)	3 (7)
II	23 (59)	17 (42)
III	14 (36)	21 (51)
**Charlson comorbidity index, median (IQR)**	6 (4–6)	6 (2–6)
**Cardiovascular comorbidities**	7 (18)	9 (22)
**Diabetes**	11 (28)	6 (15)
**Liver cirrhosis**	4 (10)	4 (10)
**Liver steatosis**	3 (8)	4 (10)
**Previous abdominal surgery**	29 (74)	26 (63)
Laparotomy	8 (21)	10 (24)
**Neoadjuvant chemotherapy**	6 (15)	5 (12)
**Multifocal lesions**	9 (23)	12 (29)
**Size of lesion, median (IQR), mm**	35 (30–49)	47 (24–80)
**Preoperative difficulty score, median (IQR)**^a^	6 (5–8)	8 (5–11)
1–5 points	15 (38)	17 (41)
6–12 points	24 (62)	24 (59)
**Diagnosis**^b^		
Primary liver malignancy	15 (39)	17 (42)
Secondary liver malignancy	24 (61)	24 (59)

Data are n (%) or median (IQR).

LH, laparoscopic hepatectomy; RH, robotic hepatectomy; ASA, American Society of Anesthesiologists; BMI, body mass index.

a Iwate difficulty scoring system is based on tumor location, tumor size, proximity to major vessels, liver function, and hand-assisted hybrid procedures.

b 5 patients in the laparoscopic hepatectomy group and 6 patients in the robotic hepatectomy group had benign lesions at final histopathology.

### Quality of life

The results of PRO data are presented in Supplemental Table S1 and Supplemental Figure S1. In each study group, three patients had at least one missing item on postoperative PRO assessments, and therefore missing PRO scores were imputed. A total of three patients in the RH-group did not respond to any postoperative PRO assessments. Of these three patients, two died and one patient was lost to follow-up. In the LH-group, one patient died and therefore did not respond to any postoperative PRO assessments. Hence, after exclusion of one patient in the LH-group and three patients in the RH-group, 38 patients in the RH group and 38 patients in the LH group remained eligible for the primary efficacy analysis of the mean role functioning scores within 90 days after surgery.

**Fig. 2:**
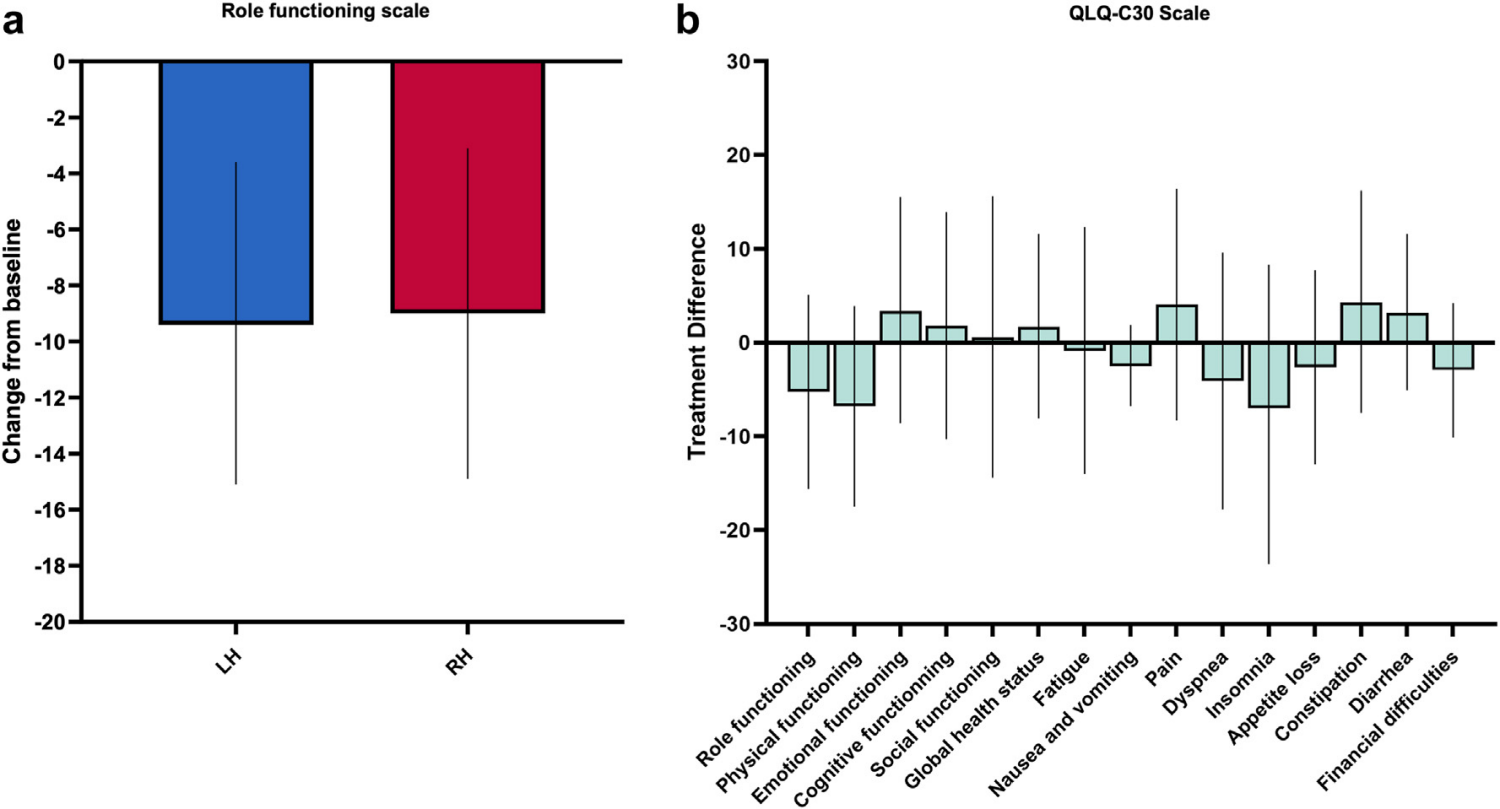
**Quality of life outcomes**. a: The mean change (bars represent mean and 95% CI) in the role functioning scores from baseline according to the QLQ-C30 questionnaire are shown in each treatment arm. There was a significant drop of role functioning scores in each treatment arm at follow-up (−9.0 (−14.9 to −3.1) versus −9.4 (−15.1 to −3.6)). The mean changes were similar in the study groups indicating no treatment effect (p = 0.547, ANCOVA with adjustments for age and baseline role functioning). b: The mean adjusted treatment differences (bars represent mean and 95% CI) of quality of life outcomes according to the QLQ-C30 questionnaire between the study groups are shown (with adjustments for age and baseline quality of life outcomes).

The analysis of the primary endpoint yielded no difference between the RH and LH groups (mean [SD], 74.3 [23.3] versus 79.6 [22.3]; p = 0.547) (Fig. 2a). Additional sensitivity analyses including adjustments for ASA status and sex, and including adjustments for ASA status, tumor size, diabetes, and difficulty score revealed no changes of the treatment effect with a mean treatment difference of −5.3 [95% CI −15.6 to 5.1] between the study groups (adjusted p = 0.417 and adjusted p = 0.370) (Supplemental Table S2). The mean treatment differences of other QLQ-C30 outcomes including functional and symptom scales ranged between −7.0 and 4.3 indicating no clinically meaningful changes between the study groups (Fig. 2b, Supplemental Table S1). In line with these data, the EQ-5D-5L outcomes also did not reveal significant and clinically meaningful treatment differences (Supplemental Figure S2). A high EQ-5D index of 0.9 points was found in both study groups (with a score of 1 indicates the best possible health state).

We performed additional analyses to address a worst-case PRO scenario in the four patients with missing PRO outcomes due to death or loss-to-follow-up. These assumptions revealed no significant differences between the RH and LH groups (mean [SD], 68.9 [29.8] versus 77.6 [25.2]) with adjustments for age and baseline role functioning score (p = 0.334), and other adjustments for baseline characteristics (sex, ASA status, tumor size, diabetes, difficulty score) with adjusted p-values of 0.335 and 0.603, respectively (Supplemental Table S3). In addition, we performed sensitivity analyses by excluding patients with benign lesions and detected no significant treatment differences between the groups (Supplemental Tables S4 and S5).

### Surgical outcomes and pathologic characteristics

Surgical details and pathologic characteristics are summarized in Supplemental Table S6. Two patients in the RH group and four in the LH group required conversion to open hepatectomy (p = 0.426), respectively. Major hepatectomy was performed for 10 patients (24%) in the RH-group and 6 patients (15%) in the LH group (p = 0.401). Of note, 1 patient in the RH group required a hepaticojejunostomy after extended right hepatectomy. The median operating time, median blood loss, extent of resection, type of procedure, location of resection, and intraoperative Iwate difficulty score were comparable between the study groups. Among patients in the RH group, two patients did not undergo resection due to significant disease progression detected at the time of surgery. R0 resection was achieved in 38 out of 39 patients (98%) in each study group (p < 0.99). Hepatocellular adenomas were the most frequent benign lesions diagnosed on final histopathology in suspected malignant lesions on preoperative imaging. Adjuvant chemotherapy was only administered in patients with cholangiocarcinoma, whereas other patients were scheduled for routine oncological follow-up.

### Safety and postoperative complications

**Fig. 3:**
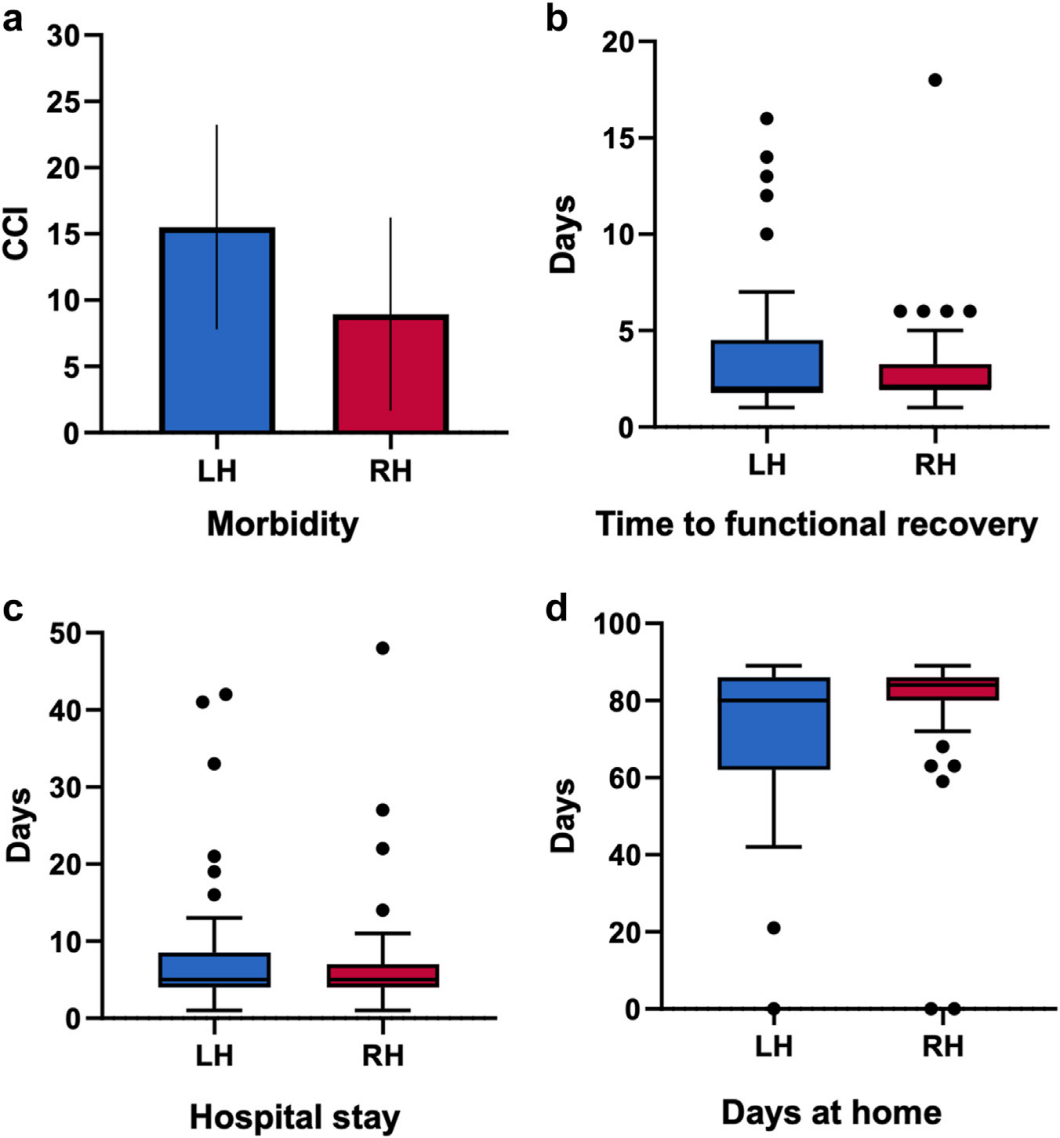
**Surgical outcomes**. a: The Comprehensive Complication Index (CCI) stratified by the study groups is displayed (bars represent mean and 95% CI). The unadjusted mean scores [standard deviation] were 8.9 [23.1] versus 15.5 [23.9] in the robotic and laparoscopic hepatectomy group, respectively. There were no significant differences between the study groups (p = 0.137, t-test). RH indicates robotic hepatectomy, LH: laparoscopic hepatectomy. b–d: Time to functional recovery, hospital stay, and days at home are presented in boxplots. No significant differences were detected between the robotic and laparoscopic hepatectomy group for the unadjusted outcomes time to functional recovery, hospital stay, and days at home (Mann–Whitney U test). For each boxplot the solid line indicates median and the box indicates interquartile range. The whiskers denote the range excluding outliers (1.5 interquartile range from the box); outliers are presented as black dots. RH indicates robotic hepatectomy, LH: laparoscopic hepatectomy.

Safety outcomes related to the intervention are shown in Table 2. A total of 10 patients (24%) in the RH group and 15 patients in the LH group (39%) developed at least one adverse event during follow-up (no patient experienced intraoperative adverse events). The most common adverse events were intraabdominal fluid collections (n = 5, 12% versus n = 10, 26%, p = 0.157) and surgical site infections (n = 3, 7% versus n = 4, 10%, p = 0.429) in the RH and LH group, respectively. Postoperative morbidity according to the Comprehensive Complication Index yielded no significant differences between the RH and LH group (8.9 [23.1] versus 15.5 [23.9], p = 0.137), respectively (Fig. 3a). Perioperative outcomes according to the Clavien-Dindo classification are detailed in Supplemental Figure S3.

**Table 2:** Safety outcomes related to intervention.

Characteristic	LH (n = 39)	RH (n = 41)	p
**Total number of documented AE**	66	23	
**Patients with at least one AE**	15 (39)	10 (24)	0.229
Pulmonary complications	4 (10)	1 (2)	0.195
Postoperative delirium	2 (5)	0	0.234
Kidney failure	5 (13)	1 (2)	0.104
Intraabdominal fluid collection	10 (26)	5 (12)	0.157
Surgical site infection	4 (10)	3 (7)	0.429
Superficial	0	1 (2)	
Deep	2 (5)	0	
Organ/Burst Abdomen	2 (5)	2 (5)	
Posthepatectomy liver failure	3 (8)	1 (2)	0.419
Grade A	1 (3)	0	
Grade B	0	0	
Grade C	2 (5)	1 (2)	
Posthepatectomy bile leakage	4 (10)	2 (5)	0.370
Grade A	0	1 (2)	
Grade B	2 (5)	1 (2)	
Grade C	2 (5)	0	
Posthepatectomy hemorrhage	4 (10)	2 (5)	0.509
Grade A	3 (8)	2 (5)	
Grade B	1 (3)	0	
Grade C	0	0	
**Interventions**	13 (33)	4 (10)	0.014
Percutaneous drain placement	8 (20)	2 (5)	0.045
Endoscopic intervention	6 (15)	0	0.011
Chest tube placement	4 (10)	1 (2)	0.195
Reintubation	1 (2)	0	0.488
Bedside wound opening	2 (5)	0	0.234
Surgical revision	5 (13)	2 (5)	0.258
**90-day mortality**	1 (3)	2 (5)	<0.99
Complication related	1 (3)	0	
Oncology related	0	2 (5)	

Data are n (%).

LH laparoscopic hepatectomy, RH robotic hepatectomy, AE, adverse events.

A total of 5 patients in the LH group had surgical revisions, of whom four patients were managed by laparotomy and one patient underwent a laparoscopic revision. In addition, two patients (who had conversion to open surgery) in the LH group were managed by bedside wound opening and vacuum-assisted wound therapy. There were two patients in the RH group were revised surgically, of whom one patient underwent laparoscopic and the other patient an open revision. There were three patients with posthepatectomy liver failure in the LH group, of whom two patients had Grade C and one patient had Grade A posthepatectomy liver failure. One patient with fatal Grade C posthepatectomy liver failure had Child B liver cirrhosis with previous portocaval shunt and underwent an anatomical segmentectomy. Another patient with Grade C posthepatectomy liver failure had extended right hepatectomy for colorectal liver metastasis and was managed on the intensive-care unit with invasive measures. However, the patient recovered completely and was discharged home on postoperative day 42. The last patient with posthepatectomy liver failure underwent right hepatectomy for hepatocellular carcinoma and developed a transient elevated INR requiring no change of clinical management (Grade A). Other posthepatectomy-specific outcomes such bile leak and hemorrhage did not differ between the study groups. The 90-day mortality rate was 5% (n = 2) in the RH group and 3% (n = 1) in the LH group (p < 0.99).

### Postoperative recovery

Details of postoperative recovery are presented in Supplemental Table S7. There were no significant differences regarding time-to-functional recovery (Fig. 3b), postoperative stay (Fig. 3c), and days at home (Fig. 3d) after discharge. The readmission rate within 90 days was 2% (n = 1) versus 13% (n = 5) in the RH and LH group (p = 0.102).

## Discussion

In this trial of adults undergoing minimally invasive hepatectomy for liver malignancies, we observed no statistically significant differences in the role functioning scale within 90 days after surgery between RH and LH. Postoperative morbidity was comparable in both groups with regard to the Comprehensive Complication Index, while the LH group required more frequent postoperative interventions. We found no significant differences in posthepatectomy-specific outcomes. Moreover, there were no observed changes in postoperative recovery or other QoL outcomes between the study groups. We further applied various imputation techniques to address missing PRO values including sensitivity analyses with adjustments for baseline demographics (i.e., ASA status, sex, tumor size, diabetes, difficulty score). None of these analyses revealed significant changes for the primary outcome. As the present study presents the first prospective study on robotic hepatectomy, the present report focusses on effectiveness and safety. Further analyses are required to evaluate the cost-effectiveness and environmental impact of RH compared to LH.

QoL after liver resection has attracted little attention in the literature as evident by lacking data from prospective trials and significant methodological flaws in PRO assessment after oncological surgery.^39^^,^^40^ To that end, previous studies assessed QoL using various questionnaires in heterogeneous cohorts who underwent primarily open hepatectomy.^39^ One randomised trial assessed QoL in minimally-invasive hepatectomy as a secondary endpoint using the generic short-form 36 (SF-36) health survey and yielded superior outcomes for LH compared to open hepatectomy.^7^ However, the SF-36 has not been developed and validated for assessment in cancer patients, and therefore misses important items on symptoms frequently experienced by oncological patients (i.e., dyspnea, appetite loss, sleep, nausea/vomiting, financial difficulties, constipation, diarrhea). Conversely, the QLQ-C30 questionnaire contains these relevant items and has been validated and established as the most widely used questionnaire in oncological patients.^31^ A previous pilot study confirmed a persistent worsening of the role functioning scale at 12 weeks after surgery as a major health outcome in patients undergoing open and laparoscopic hepatectomy.^41^ Nevertheless, prospective studies on QoL in minimally invasive liver surgery remain scarce.^28^^,^^31^^,^^39^ To the best of our knowledge, no prospective studies ever addressed QoL in patients with liver malignancies following RH. This trial is the first comprehensive report of PRO in patients undergoing RH for suspected liver malignancies. Therefore, we used the QLQ-C30 questionnaire to measure the QoL. In addition, we used a generic instrument, the EQ-5D-5L, which was primarily intended for the general population, to assess health states not covered by the QLQ-C30. Although patients developed an initial decline in their role functioning after hepatectomy, the mean QLQ-C30 outcomes reported here were still higher on follow-up than clinically important threshold values for cancer patients previously identified by others.^42^ Furthermore, we discovered that patients perceived an improved recovery within 90 days after minimally invasive hepatectomy compared to previous QLQ-C30 outcomes primarily originating from open hepatectomy.^31^ We found no treatment differences exceeding ten points in the QLQ-C30 outcomes indicating no clinically meaningful change of QoL between RH and LH.^43^ These results yielded no statistically significant differences for the primary endpoint. Notably, our PRO results of the EQ-5D-5L questionnaire revealed comparable health states before and after surgery in both study groups, including high EQ-5D index scores. Thus, our results confirm that minimally invasive hepatectomy is safe and RH represents an alternative approach to LH in patients with suspected liver malignancies.

Since the first report of RH in 2003, no prospective controlled trial to evaluate RH has yet been published.^44^ This study represents the first randomised trial investigating the impact of the robotic platform in liver surgery. Therefore, we applied broad inclusion criteria to generate high degree of generalizability by including both primary and secondary liver malignancies. However, to address the heterogeneity among patients with different kinds of liver tumors, we further stratified randomisation for primary versus secondary liver malignancies, which yielded no significant differences in the distribution of tumor entities between the study groups. Of note, despite the majority of hepatectomies in the ROC'N'ROLL trial being categorized as resections of advanced difficulty levels, the morbidity and conversion rates observed were comparable to those reported in the literature, notwithstanding the higher difficulty level and extended follow-up period of complications compared to other large cohort studies with propensity-match analyses.^17^^,^^45^, ^46^, ^47^, ^48^ Within this trial, we observed that patients undergoing RH were less likely to develop severe complications, reflected by an OR of 0.24 [95% CI, 0.07–0.84]. The majority of patients in this trial who experienced severe complications manifested intraabdominal fluid collections, pulmonary complications, posthepatectomy bile leakage or posthepatectomy liver failure, necessitating additional surgical, endoscopic or radiological interventions. None of the included patients had prophylactic intraabdominal drains except for 1 patient in the RH group who underwent a bilioenteric reconstruction. This drainage placement strategy is consistent with current trends in the literature.^49^ Historically, postoperative morbidity following hepatectomy has been remarkably high, reaching rates of up to 69% during the era of open hepatectomy.^50^, ^51^, ^52^ Advances in perioperative care and surgical techniques, particularly the increased adoption of minimally invasive approaches, have significantly reduced morbidity rates over the past decades.^53^ A meta-analysis of randomised studies comparing LH to open hepatectomy revealed a substantial decrease in morbidity (odds ratio of 0.42 [95% CI 0.30–0.58]).^54^ Another meta-analysis, including exclusively retrospective studies, indicated that RH also had a low morbidity rate compared to open hepatectomy (odds ratio of 0.67 [95% CI 0.47–0.95]).^55^ Previous comparisons between RH and LH yielded controversial conclusions on their effect on postoperative morbidity, likely due to low-level evidence with significant selection and reporting bias.^12^, ^13^, ^14^, ^15^, ^16^ Furthermore, previous studies had several limitations such as a restricted follow-up period for morbidity rates, heterogenous study populations (including or excluding malignancies), and lack of perioperative standards.^12^, ^13^, ^14^, ^15^, ^16^ In the present trial, we did not observe any difference between the oncological quality of resection in RH versus LH. Overall, we achieved a R0 resection rate in all but two patients (98%), which is at the top of the reported range from 75 to 95% in the current literature.^48^^,^^56^^,^^57^ Therefore, our results confirm the technical and oncological effectiveness of minimally invasive approaches for treating liver malignancies. This finding might help clinicians to counsel patients about outcomes regarding the choice of surgical approach.

There are some limitations to our study. First, this was a single centre trial in an academic centre with a very high expertise in minimally invasive liver surgery. During the trial period, we screened only eligible patients with suspected or confirmed liver malignancies. Hence, our results might not reflect real-world scenarios and outcomes of robotic hepatectomy could be different in centers with less experience and in patients with benign diseases. Therefore, multicenter trials with centers offering both laparoscopic and robotic hepatectomy are warranted in the future. Second, the study population included a limited number of major hepatectomies. However, as revealed by the Iwate difficulty score, the majority of patients underwent complex hepatectomies including a high number of anatomic resections as well as patients with multifocal lesions, repeat hepatectomies, and lesions located in the posterosuperior segments and caudate lobe. Third, our study aimed to assess short-term outcomes, and therefore there are no long-term outcomes reported in this trial.

### Conclusions

Among patients with suspected liver malignancies, RH yielded comparable QoL as LH and therefore can be considered a safe alternative approach at experienced centers.

## Contributors

EB designed the ROC'N'ROLL trial, wrote the first draft, and performed surgical procedures in the trial. MH, ER, and PT collected data, reviewed, and edited the manuscript. SH participated in the trial design, data analysis, and data interpretation. CR performed surgical procedures, reviewed, and edited the manuscript. NNR supervised the ROC'N'ROLL trial, reviewed and edited the manuscript, and performed surgical procedures in the trial. All authors verified the underlying data reported in the manuscript and had final responsibility for the decision to submit for publication.

## Data sharing statement

The data that support the findings of this study are available from the corresponding author upon reasonable request.

## Declaration of interests

None to be declared.

## Appendix A Supplementary data

Supplementary Figures and Tables

ROCNROLL trial protocol
