# Supplementary material for: Robotic versus laparoscopic hepatectomy for liver malignancies (ROC'N'ROLL): a single-centre, randomised, controlled, single-blinded clinical trial
Source: Lancet Reg Health Eur. 2024 Jun 24;43:100972. doi: 10.1016/j.lanepe.2024.100972 (PMC11360176; doi:10.1016/j.lanepe.2024.100972)

**Supplemental Table 1:** Patient Reported Outcomes by Intervention

|  | **Baseline** | |  | **Follow-Up** | | | |  |  |  |
| --- | --- | --- | --- | --- | --- | --- | --- | --- | --- | --- |
|  | **LH (n=38)** | **RH (n=38)** |  | **LH (n=38)** | | **RH (n=38)** | |  |  |  |
|  | **Score** | **Score** |  | **Score** | **Change from Baseline^a^** | **Score** | **Change from Baseline^a^** |  | **Adjusted Treatment Difference^b^** | |
|  | **Mean, SD** | **Mean, SD** |  | **Mean, SD** | **Mean, 95%CI** | **Mean, SD** | **Mean, 95%CI** |  | **Mean, 95%CI** | **P** |
| **Primary Endpoint** |  |  |  |  |  |  |  |  |  |  |
| Role functioning | 91·7 (15·8) | 83·3 (20·9) |  | 79·6 (22·3) | -9·4 (-15·1 to -3·6) | 74·3 (23·3) | -9·0 (-14·9 to -3·1) |  | -5·3 (-15·6 to 5·1) | 0·547 |
| **QLQ-C30 Outcomes** |  |  |  |  |  |  |  |  |  |  |
| Physical functioning | 86·8 (16·4) | 75·5 (22·9) |  | 76·1 (21·8) | -10·7 (-16·9 to -4·5) | 69·3 (25·1) | -6·2 (-12·2 to -0·2) |  | -6·8 (-17·5 to 3·9) | 0·749 |
| Emotional functioning | 64·5 (26·1) | 57·5 (28·7) |  | 68·7 (28·6) | 4·2 (-3·7 to 12·1) | 72·2 (24·0) | 14·7 (5·9 to 23·5) |  | 3·4 (-8·6 to 15·5) | 0·136 |
| Cognitive functioning | 81·1 (28·5) | 80·3 (23·8) |  | 76·2 (27·9) | -4·9 (-12·2 to -2·4) | 77·9 (24·8) | -2·3 (-8·5 to 3·9) |  | 1·8 (-10·3 to 13·9) | 0·581 |
| Social functioning | 73·2 (32·0) | 61·0 (36·8) |  | 68·0 (35·2) | -5·2 (-14·5 to 3·9) | 68·6 (30·4) | 7·6 (-2·3 to 17·5) |  | 0·6 (-14·4 to 15·6) | 0·140 |
| Global health status | 61·3 (24·8) | 58·8 (25·3) |  | 63·6 (23·1) | 2·3 (-3·5 to 8·1) | 65·3 (19·9) | 6·5 (-0·4 to 13·3) |  | 1·7 (-8·1 to 11·6) | 0·399 |
| Fatigue | 28·4 (29·9) | 38·9 (31·5) |  | 41·4 (31·4) | 13·1 (3·6 to 22·6) | 40·6 (25·9) | 1·7 (-7·4 to 10·9) |  | -0·9 (-14·0 to 12·3) | 0·257 |
| Nausea and Vomiting | 1·3 (5·9) | 6·1 (14·7) |  | 5·7 (11·6) | 4·4 (0·2 to 8·6) | 3·2 (6·9) | -2·9 (-8·1 to 2·3) |  | -2·5 (-6·8 to 1·9) | 0·235 |
| Pain | 23·7 (32·8) | 30·3 (37·3) |  | 26·8 (25·7) | 3·1 (-5·5 to 11·7) | 30·9 (28·4) | 0·6 (-7·8 to 8·9) |  | 4·1 (-8·3 to 16·4) | 0·893 |
| Dyspnea | 18·3 (29·7) | 20·1 (26·3) |  | 26·6 (32·0) | 8·2 (-1·4 to 17·8) | 22·5 (27·8) | 2·4 (-4·3 to 9·1) |  | -4·1 (-17·8 to 9·6) | 0·323 |
| Insomnia | 33·3 (39·5) | 41·2 (36·7) |  | 39·8 (35·5) | 6·4 (-2·9 to 15·7) | 32·8 (33·7) | -8·5 (-19·6 to 2·6) |  | -7·0 (-23·6 to 8·3) | 0·066 |
| Appetite loss | 16·6 (30·8) | 13·1 (21·3) |  | 17·5 (23·4) | 0·9 (-7·7 to 9·5) | 14·9 (22·1) | 1·8 (-7·9 to 11·5) |  | -2·6 (-13·0 to 7·7) | 0·763 |
| Constipation | 8·7 (20·0) | 19·2 (27·5) |  | 15·5 (25·7) | 6·8 (-0·7 to 14·3) | 19·8 (26·3) | 0·6 (-5·8 to 7·0) |  | 4·3 (-7·5 to 16·2) | 0·495 |
| Diarrhea | 11·4 (23·7) | 19·3 (27·6) |  | 11·7 (16·1) | 0·2 (-6·4 to 7·0) | 14·9 (20·1) | -4·4 (-12·5 to 3·7) |  | 3·2 (-5·1 to 11·6) | 0·905 |
| Financial Difficulties | 8·7 (22·7) | 10·5 (19·1) |  | 12·3 (18·3) | 3·5 (-1·7 to 8·7) | 9·3 (12·5) | -1·2 (-5·7 to 3·3) |  | -2·9 (-10·1 to 4·2) | 0·137 |
| **EQ-5D-5L Health States** |  |  |  |  |  |  |  |  |  |  |
| Mobility | 1·5 (0·8) | 1·5 (0·8) |  | 1·7 (0·8) | 0·2 (-0·1 to 0·4) | 1·6 (0·8) | 0·1 (-0·1 to 0·3) |  | -0·1 (-0·5 to 0.3) | 0·429 |
| Self-Care | 1·1 (0·3) | 1·2 (0·6) |  | 1·3 (0·6) | 0·2 (0·1 to 0·4) | 1·4 (0·7) | 0·2 (-0·1 to 0·4) |  | 0·1 (-0·3 to 0·3) | 0·988 |
| Usual Activities | 1·4 (0·9) | 1·8 (1·0) |  | 1·9 (1·0) | 0·5 (0·2 to 0·8) | 2·0 (1·0) | 0·2 (-0·2 to 0·6) |  | 0·1 (-0·3 to 0·5) | 0·778 |
| Pain / Discomfort | 1·8 (1·0) | 1·9 (1·0) |  | 1·8 (0·9) | 0·1 (-0·2 to 0·4) | 1·9 (0·9) | -0·1 (-0·4 to 0·2) |  | 0·1 (-0·3 to 0·4) | 0·961 |
| Anxiety / Depression | 1·6 (0·9) | 2·0 (1·0) |  | 1·6 (0·8) | 0·1 (-0·2 to 0·4) | 1·7 (0·8) | -0·4 (-0·7 to -0·1) |  | 0·1 (-0·3 to 0·3) | 0·352 |
| EQ VAS | 69·2 (20·5) | 59·5 (24·3) |  | 68·3 (21·2) | -0·8 (-6·9 to 5·3) | 67·6 (20·4) | 8·1 (0·9 to 15·3) |  | -0·7 (-10·2 to 8·8) | 0·175 |
| EQ-5D Index | 0·9 (0·2) | 0·9 (0·2) |  | 0·9 (0·1) | -0·1 (-0·2 to -0·1) | 0·9 (0·2) | 0·0 (-0·1 to 0·1) |  | 0·0 (-0·1 to 0·1) | 0·631 |

Data are mean (standard deviation) unless indicated otherwise.

LH laparoscopic hepatectomy, RH robotic hepatectomy, CI confidence interval, VAS visual analog scale, QLQ-C30 European Organisation for Research and Treatment of Cancer QLQ-C30 questionnaire, EQ-5D-5L EuroQol questionnaire

^a^ Change from baseline with positive values indicates improvement and negative values indicates deterioration in QLQ-C30 role functioning scales, QLQ-C30 global health scale, and EQ-5D-5L health states· Change from baseline with positive values indicates deterioration and negative values indicates improvement in QLQ-C30 symptom scales·

^b^ ANCOVA with adjustment for age and scores at baseline before surgery was performed

**Supplemental Table 2:** Sensitivity analysis for role functioning with adjustments for additional baseline characteristics

|  | **Baseline** | |  | **Follow-Up** | | | |  |  |  |  |  | |
| --- | --- | --- | --- | --- | --- | --- | --- | --- | --- | --- | --- | --- | --- |
|  | **LH (n=38)** | **RH (n=38)** |  | **LH (n=38)** | | **RH (n=38)** | |  |  |  |  |  | |
|  | **Score** | **Score** |  | **Score** | **Change from Baseline^a^** | **Score** | **Change from Baseline^a^** |  | **Adjusted Treatment Difference** | | | |  |
|  | **Mean, SD** | **Mean, SD** |  | **Mean, SD** | **Mean, 95%CI** | **Mean, SD** | **Mean, 95%CI** |  | **Mean, 95%CI** | **P^b^** | | **P^c^** | |
| Role functioning | 91·7 (15·8) | 83·3 (20·9) |  | 79·6 (22·3) | -9·4 (-15·1 to -3·6) | 74·3 (23·3) | -9·0 (-14·9 to -3·1) |  | -5·3 (-15·6 to 5·1) | 0·417 | | 0·571 | |

LH laparoscopic hepatectomy, RH robotic hepatectomy, CI confidence interval

^a^ Change from baseline with positive values indicates improvement and negative values indicates deterioration in QLQ-C30 role functioning scales

^b^ ANCOVA with adjustment for age, sex, ASA status and scores at baseline before surgery was performed

^c^ ANCOVA with adjustment for age, tumor size, diabetes, difficulty score, ASA status and scores at baseline before surgery was performed

**Supplemental Table 3:** Sensitivity analysis for role functioning including worst case imputation of missing patient-reported outcomes

|  | **Baseline** | |  | **Follow-Up** | | | |  |  |  |  |  | |
| --- | --- | --- | --- | --- | --- | --- | --- | --- | --- | --- | --- | --- | --- |
|  | **LH (n=39)** | **RH (n=41)** |  | **LH (n=39)** | | **RH (n=41)** | |  |  |  |  |  | |
|  | **Score** | **Score** |  | **Score** | **Change from Baseline^a^** | **Score** | **Change from Baseline^a^** |  | **Adjusted Treatment Difference** | | | |  |
|  | **Mean, SD** | **Mean, SD** |  | **Mean, SD** | **Mean, 95%CI** | **Mean, SD** | **Mean, 95%CI** |  | **Mean, 95%CI** | **P^b^** | **P^c^** | **P^d^** | |
| Role functioning | 91·9 (15·6) | 82·8 (21·0) |  | 77·6 (25·2) | -11·7 (-18·9 to -4·4) | 68·9 (29·8) | -13·9 (-22·5 to -5·8) |  | -8·7 (-21·0 to 3·6) | 0·334 | 0·335 | 0·603 | |

LH laparoscopic hepatectomy, RH robotic hepatectomy, CI confidence interval

^b^ ANCOVA with adjustment for age and scores at baseline before surgery was performed

^c^ ANCOVA with adjustment for age, sex, ASA status and scores at baseline before surgery was performed

^d^ ANCOVA with adjustment for age, tumor size, diabetes, difficulty score, ASA status and scores at baseline before surgery was performed

**Supplemental Table 4:** Sensitivity analysis for role functioning after excluding patients with benign lesions

|  | **Baseline** | |  | **Follow-Up** | | | |  |  |  |  |  | |
| --- | --- | --- | --- | --- | --- | --- | --- | --- | --- | --- | --- | --- | --- |
|  | **LH (n=33)** | **RH (n=33)** |  | **LH (n=33)** | | **RH (n=33)** | |  |  |  |  |  | |
|  | **Score** | **Score** |  | **Score** | **Change from Baseline^a^** | **Score** | **Change from Baseline^a^** |  | **Adjusted Treatment Difference** | | | |  |
|  | **Mean, SD** | **Mean, SD** |  | **Mean, SD** | **Mean, 95%CI** | **Mean, SD** | **Mean, 95%CI** |  | **Mean, 95%CI** | **P^b^** | **P^c^** | **P^d^** | |
| Role functioning | 90·4 (16·7) | 82·8 (21·0) |  | 77·9 (22·7) | -9·5 (-15·9 to -3·0) | 72·9 (23·9) | -9·8 (-16·5 to -3·0) |  | -5·0 (-16·3 to 6·5) | 0·526 | 0·374 | 0·582 | |

LH laparoscopic hepatectomy, RH robotic hepatectomy, CI confidence interval

^a^ Change from baseline with positive values indicates improvement and negative values indicates deterioration in QLQ-C30 role functioning scales

^b^ ANCOVA with adjustment for age and scores at baseline before surgery was performed

^c^ ANCOVA with adjustment for age, sex, ASA status and scores at baseline before surgery was performed

^d^ ANCOVA with adjustment for age, tumor size, diabetes, difficulty score, ASA status and scores at baseline before surgery was performed

**Supplemental Table 5:** Sensitivity analysis for role functioning after excluding patients with benign lesions including worst case imputation of missing patient-reported outcomes

|  | **Baseline** | |  | **Follow-Up** | | | |  |  |  |  |  | |
| --- | --- | --- | --- | --- | --- | --- | --- | --- | --- | --- | --- | --- | --- |
|  | **LH (n=34)** | **RH (n=35)** |  | **LH (n=34)** | | **RH (n=35)** | |  |  |  |  |  | |
|  | **Score** | **Score** |  | **Score** | **Change from Baseline^a^** | **Score** | **Change from Baseline^a^** |  | **Adjusted Treatment Difference** | | | |  |
|  | **Mean, SD** | **Mean, SD** |  | **Mean, SD** | **Mean, 95%CI** | **Mean, SD** | **Mean, 95%CI** |  | **Mean, 95%CI** | **P^b^** | **P^c^** | **P^d^** | |
| Role functioning | 90·7 (16·5) | 81·9 (21·2) |  | 75·6 (26·0) | -12·1 (-20·4 to -3·8) | 68·8 (28·8) | -13·0 (-20·9 to -5·1) |  | -6·8 (-19·9 to 6·4) | 0·542 | 0·554 | 0·552 | |

LH laparoscopic hepatectomy, RH robotic hepatectomy, CI confidence interval

^a^ Change from baseline with positive values indicates improvement and negative values indicates deterioration in QLQ-C30 role functioning scales

^b^ ANCOVA with adjustment for age and scores at baseline before surgery was performed

^c^ ANCOVA with adjustment for age, sex, ASA status and scores at baseline before surgery was performed

^d^ ANCOVA with adjustment for age, tumor size, diabetes, difficulty score, ASA status and scores at baseline before surgery was performed

**Supplemental Table 6:** Surgical Outcomes and Pathologic Characteristics

| **Characteristic** | **LH**  **(n = 39)** | **RH**  **(n = 41)** | **P** |
| --- | --- | --- | --- |
| **Conversion rate ^a, b^** | 4 (10) | 2 (5) | 0·426 |
| **Operation time, median (IQR), min** | 164 (122 – 252) | 223 (122 – 380) | 0·191 |
| **Blood loss, median (IQR), ml** | 300 (150 – 900) | 300 (100 – 500) | 0·310 |
| **Extent of resection** |  |  | 0·401 |
| Major hepatectomy | 6 (15) | 10 (24) |  |
| Minor hepatectomy | 33 (85) | 29 (71) |  |
| **Surgical procedure** |  |  | 0·923 |
| Partial hepatectomy | 12 (31) | 9 (22) |  |
| Segmentectomy | 9 (23) | 8 (21) |  |
| Left lateral sectionectomy | 5 (13) | 4 (10) |  |
| Left medial sectionectomy | 1 (3) | 0 |  |
| Right anterior sectionectomy | 1 (3) | 2 (5) |  |
| Right posterior sectionectomy | 3 (8) | 3 (7) |  |
| Segment IVb + V resection | 1 (3) | 1 (2) |  |
| Segment V + VI resection | 1 (3) | 1 (2) |  |
| Segment VIII + IVa + III resection | 0 | 1 (2) |  |
| Right posterior + left central sectionectomy | 0 | 1 (2) |  |
| Central hepatectomy | 1 (3) | 2 (5) |  |
| Left (extended) hepatectomy | 2 (5) | 3 (7) |  |
| Right (extended) hepatectomy | 3 (8) | 4 (10) |  |
| No resection performed | 0 | 2 (5) |  |
| **Location of resection** |  |  |  |
| Segment I | 2 (5) | 2 (5) | <0·99 |
| Segment II | 15 (39) | 10 (24) | 0·229 |
| Segment III | 9 (23) | 12 (29) | 0·615 |
| Segment IVa | 7 (17) | 8 (20) | <0·99 |
| Segment IVb | 9 (23) | 9 (22) | <0·99 |
| Segment V | 14 (36) | 15 (37) | <0·99 |
| Segment VI | 14 (36) | 12 (29) | 0·635 |
| Segment VII | 12 (31) | 10 (24) | 0·619 |
| Segment VIII | 8 (21) | 14 (34) | 0·619 |
| **Intraoperative difficulty score, median (IQR)^c^** | 6 (6 – 9) | 9 (6 – 11) | 0·346 |
| **Pathology** |  |  | 0·436 |
| Cholangiocarcinoma | 1 (3) | 5 (12) |  |
| Hepatocellular carcinoma | 10 (26) | 6 (15) |  |
| Colorectal liver metastasis | 16 (41) | 18 (44) |  |
| Other malignancies **^d^** | 7 (18) | 6 (15) |  |
| Hepatocellular adenoma | 3 (8) | 3 (7) |  |
| Other benign lesions **^e^** | 2 (5) | 3 (7) |  |
| **Negative resection margin ^f^** | 38/39 (98) | 38/39 (98) | <0·99 |
| **Tumor stage** |  |  | 0·846 |
| T1 / T2 | 9 (23) | 11 (27) |  |
| T3 / T4 | 2 (5) | 1 (2) |  |
| Not available | 27 (72) | 29 (71) |  |
| **Nodal status ^g^** |  |  | <0·99 |
| Negative | 0 | 2 (5) |  |
| Positive | 1 (3) | 2 (5) |  |
| Not available | 38 (98) | 37 (90) |  |
| **Vascular invasion** |  |  | 0·084 |
| Negative | 9 (23) | 7 (17) |  |
| Positive | 0 | 5 (12) |  |
| Not available | 30 (77) | 29 (71) |  |
| **Perineural invasion** |  |  | 0·791 |
| Negative | 7 (18) | 10 (24) |  |
| Positive | 1 (3) | 1 (2) |  |
| Not available | 31 (79) | 30 (73) |  |
| **Grading** |  |  | 0·165 |
| G1 / G2 | 10 (26) | 8 (29) |  |
| G3 / G4 | 0 | 4 (10) |  |
| Not available | 29 (74) | 29 (71) |  |

Data are n (%) or median (IQR)

LH laparoscopic hepatectomy, RH robotic hepatectomy,

^a^ conversion in the LH group due to oncological reasons (anatomic segment VIII resection for recurrent hepatocellular carcinoma (n=1)); and due to adhesions (parenchyma-sparing segment VII resection for ovarian carcinoma metastasis (n=1); anatomic segment V/VI resection for synchronous colorectal liver metastasis with right colectomy (n=1), and left medial sectionectomy for recurrent colorectal liver metastasis (n=1))

^b^ conversion in the RH group due to oncological reasons (extended left hepatectomy for a centrally located hepatocellular carcinoma (n=1)) and due to adhesions (left lateral sectionectomy for recurrent colorectal liver metastasis(n=1))

^c^ Iwate difficulty scoring system is based on tumor location, tumor size, proximity to major vessels, liver function, and hand-assisted hybrid procedures

^d^ Other malignancies in the laparoscopic hepatectomy group were as following: n=1 breast cancer liver metastasis, n=1 ovarian cancer liver metastasis, n=1 thymus cancer liver metastasis, n=1 neuroendocrine liver metastasis, n=2 renal cell carcinoma liver metastasis, n=1 non-small cell lung cancer liver metastasis; other malignancies in the robotic hepatectomy group were as following: n=2 breast cancer liver metastasis, n=1 melanoma liver metastasis, n=1 neuroendocrine liver metastasis, n=1 renal cell carcinoma liver metastasis, n=1 primary sarcoma

^e^ Other benign lesions in the laparoscopic hepatectomy group were as following: n=1 focal nodular hyperplasia, n=1 hydatid cyst (multilocular); other benign lesions in the robotic hepatectomy group were as following: n=2 focal nodular hyperplasia, n=1 angiomyolipoma

^f^ Resection status was available for 39 patients in the laparoscopic hepatectomy group and 39 patients in the robotic hepatectomy group

^g^ Routine hilar lymphadenectomy was only performed in patients with suspected cholangiocarcinoma.

**Supplemental Table 7:** Posthepatectomy recovery

| **Characteristic** | **LH**  **(n = 39)** | **RH**  **(n = 41)** | **P** |
| --- | --- | --- | --- |
| **Time-to-functional recovery, median (IQR, days** | 2 (2 – 4) | 2 (2 – 3) | 0·671 |
| **Length of stay, median (IQR), days** | 5 (4 – 9) | 5 (4 – 7) | 0·421 |
| **Days at home after discharge, median (IQR), days** | 80 (63 – 86) | 84 (81 – 86) | 0·094 |
| **Readmission to hospital < 90 days** | 5 (13) | 1 (2) | 0·102 |

Data are n (%) or median (IQR)

LH laparoscopic hepatectomy, RH robotic hepatectomy

**Supplemental Figure 1: QLQ-C30 outcomes**

The mean change (bars represent mean and 95%CI) according to the QLQ-C30 questionnaire are shown. The QLQ-C30 outcomes were adjusted for age and baseline QLQ-C30 value and compared between the study groups using ANCOVA. No significant differences were detected for the mean changes between the study groups indicating no treatment effect. Higher scores on functional and global health score indicate better quality of life, while higher scores on symptom scales indicate worse quality of life. RH indicates robotic hepatectomy (red colour), LH: laparoscopic hepatectomy (blue colour).


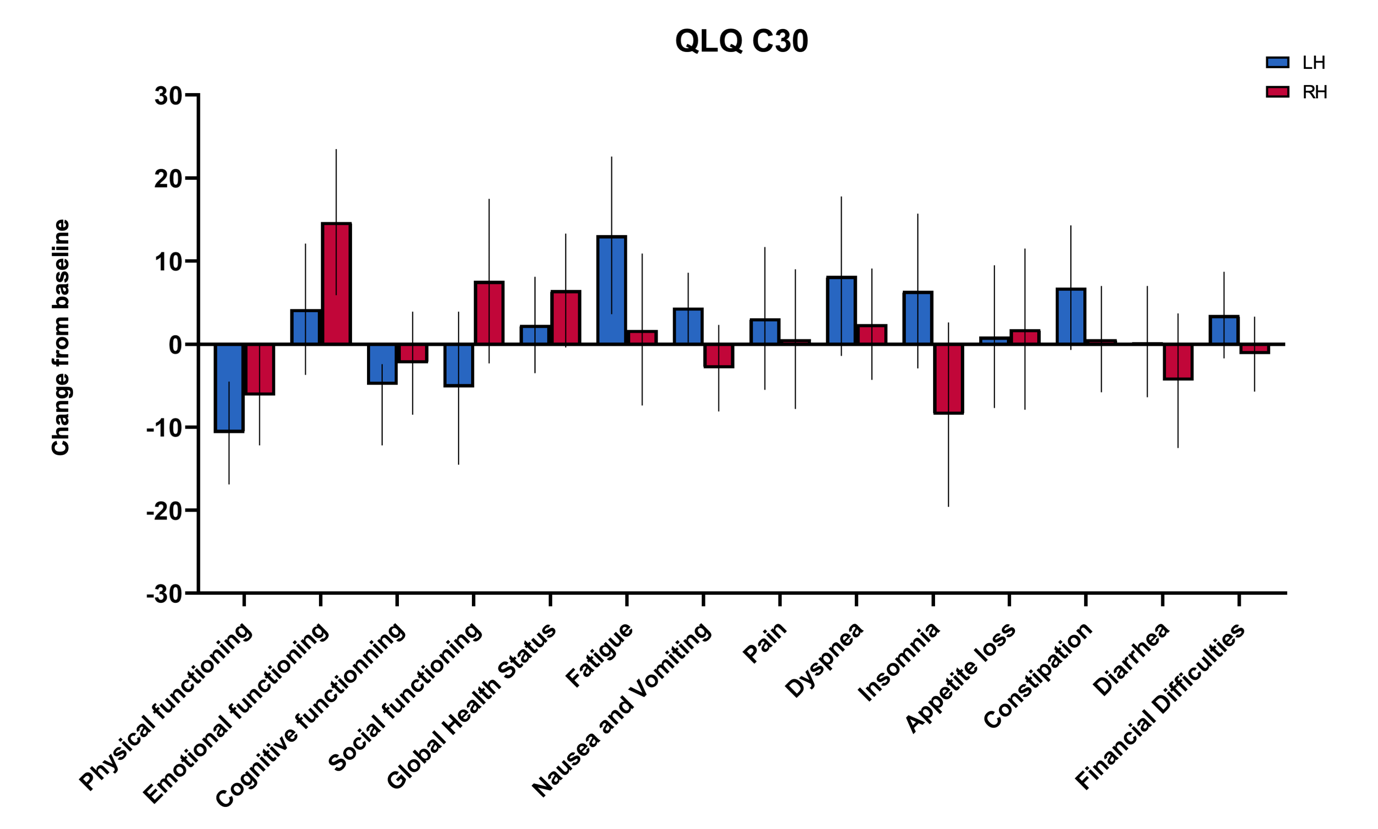


**Supplemental Figure 2: EQ-5D-5L outcomes**

A-C: The mean change (bars represent mean and 95%CI) of EQ-5D-5L health states, EQ-5D-5L index values and EQ-5D-5L VAS score from baseline are shown, respectively. The EQ-5D-5L outcome were adjusted for age and baseline EQ-5D-5L values and compared between the study groups using ANCOVA. No significant differences were detected. An index score of 1 represent the best possible health status, and the VAS score ranges from 0-100 with higher scores indicating better quality of life. RH indicates robotic hepatectomy, LH: laparoscopic hepatectomy


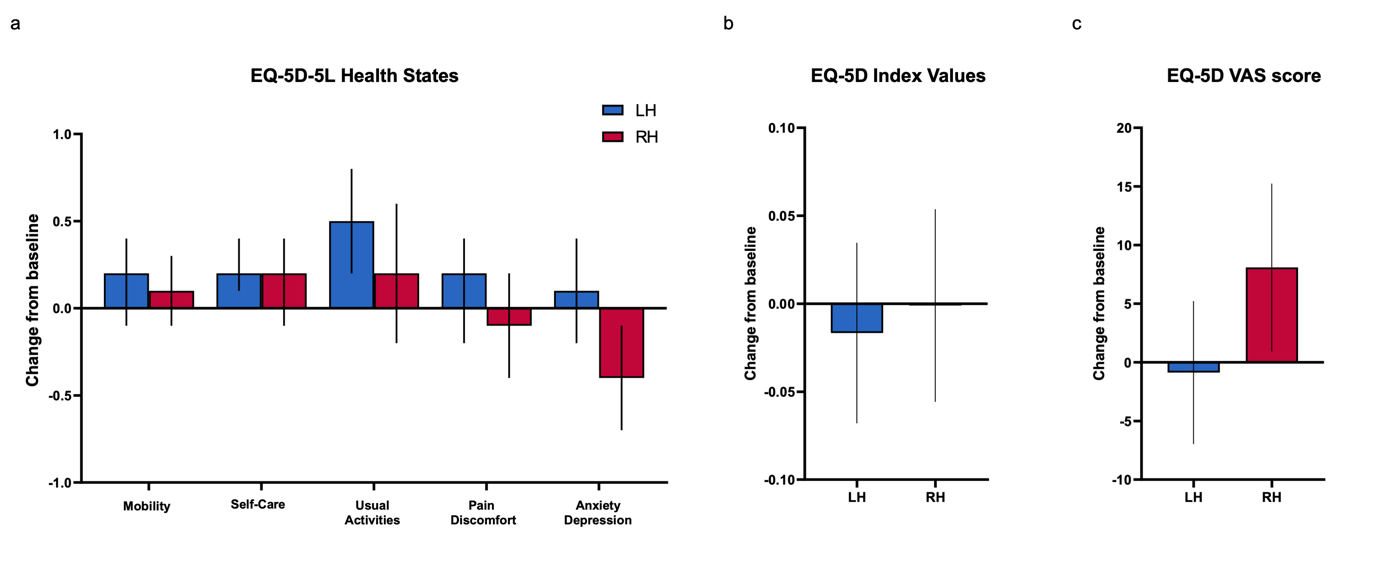


**Supplemental Figure 3:**

The distribution of postoperative complications stratified by the study groups are displayed (graded by the Clavien-Dindo classification). Data are reported in numbers (n) and frequencies (%). A total of 10 patients in the robotic hepatectomy group and a total of 15 patients in the laparoscopic hepatectomy group had at least one adverse event (n=10, 24% vs. n=15, 38%, p = 0·229, Fisher's exact test).


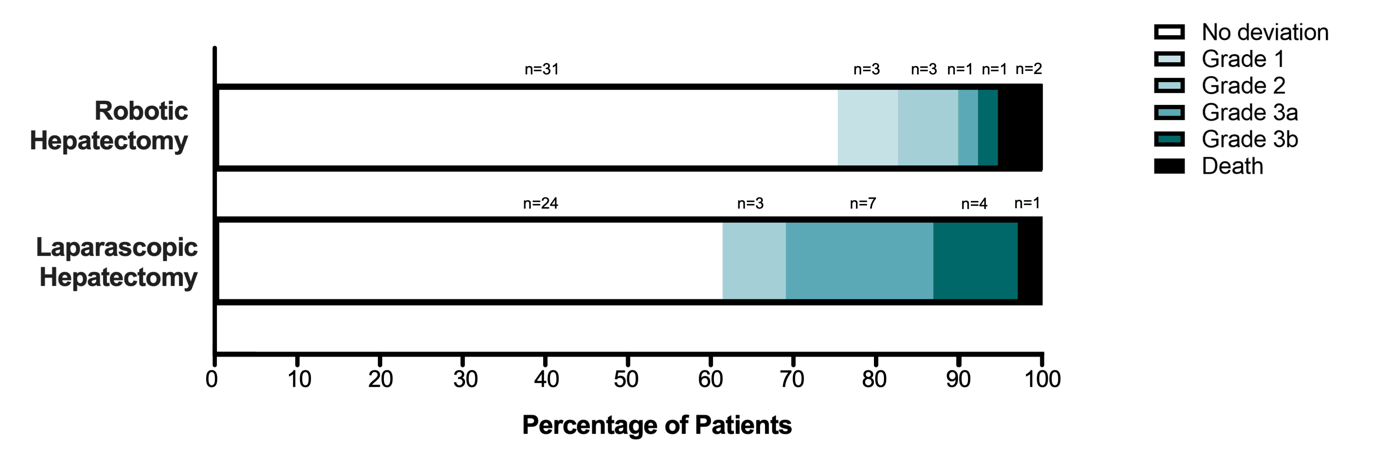

Supplement: Supplementary Figures and Tables [file mmc1.docx]
