# Supplementary material for: Robotic versus laparoscopic hepatectomy for liver malignancies (ROC'N'ROLL): a single-centre, randomised, controlled, single-blinded clinical trial
Source: Lancet Reg Health Eur. 2024 Jun 24;43:100972. doi: 10.1016/j.lanepe.2024.100972 (PMC11360176; doi:10.1016/j.lanepe.2024.100972)
Supplement: ROCNROLL trial protocol [file mmc2.pdf]

Roc’n’Roll

Role functioning in Robotic assisted versus laparoscopic liver resection

The information in this trial protocol is strictly confidential. It is for the use of the investigator, trial personnel, ethics committee, the authorities, and trial subjects only. This trial protocol may not be passed on to third parties without the agreement of the principal investigators.

Protocol written by:

Emrullah Birgin

ROLES AND RESPONSIBILITIES

Principal Investigators

PD Dr. Emrullah Birgin, MD

Heidelberg University
Medical Faculty Mannheim
Department of Surgery
Theodor-Kutzer-Ufer 1-3
68167 Mannheim, Germany
Phone: +49-621-383-3671
Fax: +49-621-383-3809
Email: emrullah.birgin@umm.de

Prof. Dr. Nuh N. Rahbari, MD

Heidelberg University
Medical Faculty Mannheim
Department of Surgery
Theodor-Kutzer-Ufer 1-3
68167 Mannheim, Germany
Phone: +49-621-383-3591
Email: nuh.rahbari@umm.de

Biostatisticians

Dr. sc. hum. Svetlana Hetjens

Department of Medical Statistics, Biomathematics and Informatics
Theodor-Kutzer-Ufer 1-3
68167 Mannheim, Germany
Phone: +49-621-383-9902
Email: svetlana.hetjens@medma.uni-heidelberg.de

Study Monitor

Dipl. Biol. Dorothée Sturm

Heidelberg University
Medical Faculty Mannheim
Department of Surgery
Theodor-Kutzer-Ufer 1-3
68167 Mannheim, Germany
Email: dorothee.sturm@umm.de
Phone: +49-621-383-4864
Fax: +49-621-383-2109

Investigator

PD Dr. Patrick Téoule, MD

Heidelberg University
Medical Faculty Mannheim
Department of Surgery
Theodor-Kutzer-Ufer 1-3
68167 Mannheim, Germany
Email: patrick.téoule@umm.de
Phone: +49-621-383-2756

TABLE OF CONTENTS

ROLES AND RESPONSIBILITIES 2
PROTOCOL OUTLINE 4
ABBREVIATIONS 6
1 INTRODUCTION 7
2 TRIAL OBJECTIVES AND ENDPOINTS 8
3 TRIAL DESIGN 14
4 STANDARDIZATION OF CARE 15
5 TRIAL INTERVENTIONS 17
6 TRIAL DURATION AND SCHEDULE 18
7 SELECTION OF SUBJECTS 19
8 CRITERIA FOR PATIENT WITHDRAWAL AND STUDY TERMINATION 20
9 METHODS AGAINST BIAS 21
10 TRIAL IMPLEMENTATION 22
11 ASSESSMENT OF SAFETY 23
12 STATISTICAL CONSIDERATIONS 24
13 DATA MANAGEMENT 25
14 ETHICAL CONSIDERATIONS 26
15 REGULATORY OBLIGATIONS 27
REFERENCES 28
SIGNATURES 30
TRIAL FLOW CHART 31
APPENDIX A: QLQ-C30 QUESTIONNAIRE 32
APPENDIX B: DIFFICULTY SCORING SYSTEM 34
APPENDIX B: EQ-5D-5L 35
APPENDIX D: CLASSIFICATION OF SURGICAL COMPLICATIONS 37
APPENDIX E: MOBILITY SCALE 38

PROTOCOL OUTLINE

Title (Acronym)

Roc'n'Roll: Role functioning in Robotic-assisted versus laparoscopic liver resection

Study population

Patients scheduled for minimally invasive surgery for primary or secondary liver malignancies

Objective

To assess the average quality of life (QoL) during 90 days after surgery, measured 30 days, 60 days, and 90 days after surgery by the EORTC QLQ-C30 scale role functioning (RF)

Trial Design

Prospective, randomized controlled, open superiority trial with two parallel study groups

Population

Inclusion criteria:

- Age equal to or greater than 18 years
- Curative-intent minimally invasive (robotic-assisted or laparoscopic) liver surgery for confirmed or suspected primary or secondary malignancies
- Written informed consent
- Ability and willingness to respond questionnaires

Exclusion criteria:

- ASA ≥ 4
- Impaired mental state or inability of understanding the German language (no fluent German language speaker)
- Expected lack of compliance

Study intervention

Robotic-assisted liver resection

Control intervention

Laparoscopic liver resection

Sample Size

Expected to be assessed for eligibility (n = 200)

Expected to be allocated to trial (n = 90)

Expected to be analyzed (n = 76)

Statistical Analysis

Primary efficacy endpoint: Average level of the QLQ-C30 scale “role functioning” at 90 days after surgery

Description of the primary efficacy analysis and population: The sample size calculation is based on the primary endpoint and a two-sided t-test. The confirmatory analysis is performed on the basis of an intention-to-treat (ITT) population and with respect to ITT principles. The level of significance is set at 5% (two-sided) and the sample size is determined to assure a power of $1-\beta=80\%$

Safety: Exploratory analyses of frequencies of complications and clinical events

Secondary endpoints: Type and extent of liver resection, Difficulty of liver resection, Operating time, Total blood loss, Conversion rate to open surgery, Time-to-functional recovery, Postoperative hospital stay, Postoperative intensive/intermediate care unit stay, Readmission to intermediate/intensive care unit, Readmission rate to hospital, Need for invasive interventions, Frequency of postoperative medical complications, Frequency of postoperative surgical complications, Rate of perioperative morbidity, Pathological characteristics, Days in home environment, Discharge destination from hospital, Return to oncologic intended therapy, HRQoL, Costs

Trial Duration and Dates

First patient in to last patient out: February 2022 to May 2024

Duration of the entire trial: 27 months

ABBREVIATIONS

**AE**	Adverse Event
**ANCOVA**	Analysis of covariance
**ASA**	American Society of Anesthesiologists
**CRF**	Case report form
**CTCAE**	Common terminology criteria for adverse events
**CVP**	Central venous pressure
**eCRF**	Electronic case report form
**EORTC**	European organization for research and treatment of cancer
**ERCP**	Endoscopic retrograde cholangiopancreaticography
**GCP**	Good Clinical Practice
**HRQoL**	Health-related quality of life
**ICH**	International Conference on Harmonization of Technical Requirements for Registration of Pharmaceuticals for Human Use
**ICMJE**	International Committee of Medical Journal Editors
**ICU**	Intensive Care Unit
**IEC**	Independent Ethics Committee
**IMU**	Intermediate Care Unit
**ITT**	Intention-to-treat
**MILS**	Minimally invasive liver surgery
**POD**	Postoperative day
**PROMs**	Patient-reported outcome measures
**QALY**	Quality-adjusted life years
**QoL**	Quality of Life
**SDV**	Source data verification

1 INTRODUCTION

1.1 Scientific Background

Liver surgery remains the only curative treatment for patients with primary or secondary liver malignancies. However, liver surgery is associated with a high risk for postoperative complications which results in poor postoperative recovery and quality of life.[1-5] From patient´s perspective, however, being able to maintain one’s usual life after cancer surgery is crucial. Although the assessment of the patient´s ability to fulfill different roles in their daily life (e.g., to be able to return to work, to care for oneself, to participate in social activities) is quite complex, role functioning is a core construct and major focus in health outcome research in oncology.[6,7] Patient-reported outcome measures (PROMs) enable the measurement of patients’ symptom burden and role functioning after cancer treatment.[8] The most widely used validated questionnaire in oncology to assess HRQoL is the EORTC QLQ-C30.[9] After surgery, patient´s health-related quality of life (HRQoL) is usually impaired in the first 3 months.[10,11]

In surgical oncology, strategies to improve HRQoL are urgently needed but high-quality data from prospective studies remain scarce. In recent years, minimally invasive liver surgery (MILS) has been increasingly used for treatment of liver malignancies.[12] However, there is currently no randomized controlled trial existing to compare the HRQoL as primary outcome in patients treated by MILS for primary and secondary liver malignancies. One randomized controlled trial comparing laparoscopic liver resections and open liver resections in colorectal liver metastases demonstrated lower postoperative complications and better postoperative HRQoL in patients treated laparoscopically.[5,13] However, HRQoL was assessed as secondary outcome with the Short Form 36 questionnaire, which has not been primarily designed and validated for oncological patients. In MILS, robotic-assisted surgery is an emerging specialty which allows surgeons to perform more precise movements with enhanced dexterity and delicate dissection compared to conventional laparoscopic techniques.[14,15] The robotic system offers an improved visualization without the need for an additional assistant to hold the camera, a comfortable operating position for the operating surgeon, eliminates unnatural postures, and suppresses tremor associated with conventional laparoscopy. Complex liver resections, advanced hilar dissections, and curved parenchymal dissections might be more accessible for robotic surgery compared to laparoscopic surgery. Thus, a more precise surgery could result in enhanced recovery of patients and improved HRQoL after liver resection.

Unfortunately, the current evidence comparing laparoscopic versus robotic liver resections is only limited on cohort studies. These studies show a high selection bias with inconclusive results regarding postoperative outcomes while there is no data extending on HRQoL after robotic surgery.[16-19] Taken together, advantages of robotic-assisted surgery might result in a more precise surgery with enhanced recovery of patients and improved HRQoL after liver resection.

1.2 Aim of the trial

The Roc’n’Roll trial aims to compare the postoperative HRQoL during 90 days after surgery by the EORTC QLQ-C30 role functioning scale after laparoscopic and robotic-assisted liver resection. Furthermore, surgical characteristics outcomes, pathological outcomes, perioperative morbidity, and costs of both surgical techniques will be evaluated as secondary outcomes.

2 TRIAL OBJECTIVES AND ENDPOINTS

Primary Efficacy Objective

To assess the average quality of life (QoL) during 90 days after surgery, measured 30 days, 60 days, and 90 days after surgery by the EORTC QLQ-C30 scale role functioning (RF)

2.1 Primary endpoint

The average QoL during 90 days after surgery by the EORTC QLQ-C30 RF scale

2.2 Secondary endpoints

- Type and extent of liver resection
- Difficulty of liver resection
- Operating time
- Total blood loss
- Conversion rate to open surgery
- Time-to-functional recovery
- Postoperative hospital stay
- Postoperative intensive/intermediate care unit stay
- Readmission to intermediate/intensive care unit
- Readmission rate to hospital
- Need for invasive interventions
- Frequency of postoperative medical complications
- Frequency of postoperative surgical complications
- Rate of perioperative morbidity
- Pathological characteristics
- Days in home environment
- Discharge destination from hospital
- Return to oncologic intended therapy
- HRQoL
- Costs

2.3 Assessment of endpoints

2.3.1 Average QoL during 90 days after surgery by the EORTC QLQ-C30 "RF" scale

The German version of the standardized questionnaire QLQ-C30[20] (version 3.0) of the European Organization for Research and Treatment of Cancer (EORTC) will be used (see Appendix A) to measure the mean QoL using the RF scale (range: 0-100). The RF will be evaluated before surgery, and at postoperative days 30, 60, and 90. If the patient is willing to respond the survey on follow-up by email or telephone, this will be separately documented.

2.3.2 Type and extent of liver resection

The type of liver resection will be documented as anatomic or non-anatomic liver resection and the extent of resection as major (defined as anatomic resection of >3 liver segments) or minor liver resection, respectively.

2.3.3 Difficulty of liver resection

The difficulty of liver resection will be assessed by the IWATE-criteria with following six difficulty indices before surgery[21] (Appendix B): 1. Tumor location, 2. Extent of liver resection, 3. Tumor size, 4. Proximity to major vessel, 5. Liver function, 6. Hybrid technique. A scoring system 0-12 will be used and scores >6 will be defined as "difficult".

2.3.4 Operating time [min]

Time from skin incision until placement of last skin staple/suture.

2.3.5 Total blood loss [ml]

Intraoperative blood loss will be measured according to the blood collected in the suction containers. Spilling water and ascites will be subtracted. Furthermore, swabs will be squeezed, and their content will also be sucked and added to the fluid collected in the suction containers.

2.3.6 Conversion rate to open surgery

The conversion rate to open liver resection will be documented.

2.3.7 Time-to-functional recovery [days]

The postoperative functional recovery is achieved if the following four criteria are fulfilled:

1) Pain control with oral medication (numeric rating scale <4);
2) Solid food intake
3) No need for intravenous fluids
4) Independence in mobility or equal to the preoperative level

The following outcomes will be additionally recorded:

a) *Time to pain control with oral medication [days];*
b) *Time to food intake [days]:* sips of water, semi-fluid diet, soft bland diet;
c) *Time to bowel movement [days]:* first flatus, first bowel movement;

d) *Time to relief of intravenous fluids [days];*

e) *Time to independent mobility or level of preoperative level [days]*: Preoperative level of the mobility scale[22] or a positive score for 8 of 10 items (see Appendix E).

2.3.8 Postoperative hospital stay [days]

Time from the day of operation until the day of discharge.

2.3.9 Postoperative intermediate/intensive care unit stay [days]

Days on the intermediate care unit (IMU or intensive care unit (ICU) after surgery. Patient´s stay in the recovery room exceeding 24 hours is counted as ICU stay.

2.3.10 Readmission to intermediate/intensive care unit stay

Postoperative readmission to the intermediate/intensive care unit until POD 90

2.3.11 Readmission rate

Unplanned readmission at hospital within 90 days after surgery

2.3.12 *Need for invasive interventions*

Invasive interventions such as placement of interventional drains, endoscopic retrograde cholangiopancreaticography (ERCP) with stent placement, chest tube placement, invasive ventilation, and re-laparotomy will be documented up to 90 days after surgery.

2.3.13 *Postoperative medical complications*

The following set of predefined complications will be considered as postoperative non-surgical clincal events and documented in the Roc’n’Roll-Trial in line with the definitions of the common terminology criteria for adverse events version 5.0 if not otherwise specified:

Cardiovascular complications: Myocardial infarction (necrosis of the myocardium due to an interruption of blood supply), Stroke (decrease or absence of blood supply to the brain caused by obstruction (thrombosis or embolism) of an artery resulting in neurological damage), *Deep venous thrombosis (formation of a blood clot in large veins) Pulmonary complications*: Pulmonary infection[23], Atelectasis[24], Acute Respiratory Distress Syndrome[25], *Pulmonary embolism (*characterized by occlusion of the pulmonary vessels by a thrombus that has migrated from a distal site via the blood stream)

Postoperative delirium[26]

Acute kidney insufficiency[27].

2.3.14 *Postoperative surgical complications*

The following set of predefined complications will be considered as postoperative surgical clinical events and documented in the Roc’n’Roll-Trial in line with the definitions of the common terminology criteria for adverse events version 5.0 if not otherwise specified:

Surgical site infection according to the Centers for Disease Control and Prevention grading: superficial, deep, organ/space

Intraabdominal fluid collection/abscess: Intraabdominal fluid collection detected on any imaging modality (e.g. ultrasound, CT scan) associated with abdominal discomfort/pain and/or elevation of infectious parameters.

Posthepatectomy complications (Grade A-C) according to the International Study Group of Liver Surgery[9,10,28]: Posthepatectomy hemorrhage, Postoperative biliary leakage, Posthepatectomy liver failure.

2.3.15 *Perioperative morbidity*

Perioperative morbidity and mortality will be documented according to the Clavien-Dindo-Classification until 90 days after surgery[29].

2.3.16 *Pathological characteristics*

Pathological characteristics including tumor size, nodal status, margin status, and lymphvascular invasion will be documented as provided by the pathological report.

2.3.17 Days in home environment 30 days and 90 days of surgery[30] [days]

Days in home environment is calculated from the date of surgery using hospitalization and mortality data. Death of a patient in hospital or after discharge within 30 days (or 90 days) will be assigned as “0”. A discharge in a postdischarge nursing facility will not be counted as days at home.

2.3.18 Discharge destination from hospital

Discharge destination will be classified as home, nursing home, acute-care hospital, and rehabilitation facility.

2.3.19 Oncologic intended therapy

The return to intended oncologic therapy following surgery will be recorded at postoperative day 90.

2.3.20 HRQoL

The QLQ-C30[20] and the EQ-5D-5L questionnaire will be used to assess the HRQoL (see Appendix A and C). All questionnaires will be evaluated before surgery, and at postoperative days 30, 60, and 90. If the patient is willing to respond the survey on follow-up by email or telephone, this will be separately documented.

2.3.21 Costs

Economic evaluation will be performed as cost-utility analyses using quality-adjusted life years (QALY) as endpoint with a time horizon of 90 days.[31] The use of hospital healthcare resources will be collected from the case report forms and clinical information system. The following resources will be assessed: duration of admission on general ward and intensive/intermediate care unit, surgical approach, radiological and endoscopic procedures, outpatient clinic visits, emergency room visits, and readmissions. The unit costs of

laparoscopic and robotic-assisted liver resections will be calculated from costs of operating theatre use, personnel, purchase of disposable materials, and overhead costs. QALYs wil be assessed using the EQ-5D data and the German tariff.[32] The incremental cost–utility ratio will be calculated as the difference in mean costs per patient divided by the difference in mean QALYs per patient.

3 TRIAL DESIGN

This is a prospective randomized controlled, open superiority trial with two parallel study groups. The trial is designed to show that the average HRQoL during 90 days after surgery by the EORTC QLQ-C30 “RF” scale is higher in patients with robotic-assisted liver surgery compared to patients with laparoscopic liver surgery.

The following hypothesis will be tested:
H0: No difference between the study groups in the mean of the primary variable “role functioning”
H1: “Role functioning” is different between the study groups.

Patients scheduled for curative-intent minimally invasive (robotic-assisted or laparoscopic) liver surgery for confirmed or suspected primary or secondary malignancies will be screened for inclusion into the trial. Patients meeting the above-mentioned eligibility criteria will be enrolled into the study. Written informed consent is obtained at least on the day before surgery.

4 STANDARDIZATION OF CARE

Patients' perioperative care will be standardized and kept identical except for the approach of surgery in the study groups (laparoscopic vs. robotic-assisted liver resection). Patients are treated in line with the German S3 guidelines and established clinical pathways of the Department of Surgery, University Medical Center Mannheim. The clinical pathways are outlined as "Standard Operating Procedures" in the document database "roxtra" of the Department of Surgery.

Standardization of anesthesiological care

Patients are equipped with 16 or 18 Gauge peripheral venous catheter or central venous catheter depending on individual patient´s comorbidities. Patients are treated with general anesthesia receiving antibiotic prophylaxis (2g cefuroxime and 500mg metronidazole, or clindamycin 600mg in case of allergy against cefuroxime respectively gentamicin 3mg/kg body weight in case of allergy against metronidazole) within 30-60 minutes prior to incision. General anesthesia is induced using Thiopental (3-5mg/kg body weight) or Propofol (1-2mg/kg body weight), additional Sufentanil (0.2-0.4mg/kg body weight) and Cisatracurium (0.15mg/kg body weight) or Rocuronium (0.6mg/kg body weight). Anesthesia is maintained using either Sevoflurane (minimal alveolar concentration 0.8) or Desflurane (minimal alveolar concentration 0.8) and additional Sufentanil (up to 1µg/kg body weight total). All drugs are administered adapted to the patient´s body weight. Patients are equipped with gastric tube, arterial catheterization, 16 Gauge peripheral venous catheter, twin lumen central venous catheter (7 French; 18/14 Gauge) or shock catheter (12 French; 12/16/18 Gauge), and transurethral (or suprapubic if contraindicated) catheter with temperature probe, respectively. Intravenous fluid therapy and administration of blood prodcuts will be performed according to local standards aiming a central venous pressure (CVP) below 5mmHg.[43]

Standardization of surgical techniques

Only surgeons with experience of >50 robotic or laparoscopic hepatectomies per year and >25 robotic hepatectomies will be allowed to perform operations in this trial. A CO_2 pneumoperitoneum of 12mmHg is established in each group after placing the trocars. Initially, the abdomen is explored for extrahepatic disease. Intraoperative ultrasound of the liver is carried out routinely to exclude previously undetected lesions and to determine the resection planes. A tape is placed around the hepatoduodenal ligament for subsequent intermittent Pringle maneuver during parenchymal transection.

In all groups parenchymal transection is carried out under the following settings:

- CO_2 pneumoperitoneum of 15-18 mmHg;
- Reversed Trendelenburg position;
- Positive end-expiratory pressure (PEEP) of 0 mmHg;
- Intermittent Pringle maneuver (10 minutes of ischemia followed by 5 minutes of reperfusion).

Parenchymal transection is carried out using the crush-clamp technique in combination with an energy device (LigaSure, Covidien MedTronic or Thunderbeat, Olympus) or bipolar forceps. Vascular and biliary structures with a diameter of >2 mm are divided using titan (Braun) or Hem-o-Lock (VECK) clips. Major pedicles and hepatic veins are divided using Autosuture Endo Gia™ Universal Stapler and Endo Gia™ Universal Angulating 45 mm loading units with 2.5 mm staples (Covidien).

- Laparoscopic group: Four trocars are used (1x 12 mm, 1x 10 mm, 2x 5 mm) in a standardized fashion. Parenchymal transection is carried out using the settings as described above.
- Robotic-assisted group: Five trocars are used (1x 12 mm, 4x robotic trocars) in a standardized fashion. Parenchymal transection is carried out using the settings as described above.

Standardization of postoperative care

Patients are transferred either to the post-anesthesia care unit and regular surgical ward, or directly to the intermediate-care unit/intensive-care unit depending on patient´s comorbidities as well as the intraoperative course. In both treatment arms, the postoperative care is identical adhering to enhanced recovery after surgery principles as outlined in the clinical pathways of the Department of Surgery.

5 TRIAL INTERVENTIONS

Patient´s perioperative care will be standardized and kept identical except for the approach of surgery, i.e. laparoscopic versus robotic-assisted liver surgery.

5.1 Control Group (Group A)

Patients randomized to the laparoscopic group will undergo laparoscopic liver resection.

5.2 Experimental Group (Group B)

Patients randomized to the experimental group will undergo robotic-assisted liver resection.

6 TRIAL DURATION AND SCHEDULE

Recruitment of patients is expected to last 24 months. The first patient and the last patient will be enrolled in February 2022 and February 2024, respectively.

**First patient in:**	**February 2022**
**Last patient in:**	**February 2024**
**Last patient out:**	**May 2024**
**Database Closure:**	**June 2024**
**Statistical Analysis:**	**July 2024**
**Report:**	**August 2024**

7 SELECTION OF SUBJECTS

Subjects matching the following criteria are eligible for inclusion into the clinical trial:

Inclusion criteria:

- Age equal to or greater than 18 years
- Curative-intent minimally invasive (robotic-assisted or laparoscopic) liver surgery for confirmed or suspected primary or secondary malignancies
- Written informed consent
- Ability and willingness to respond questionnaires

Exclusion criteria:

- ASA ≥ 4
- Impaired mental state or inability of understanding the German language (no fluent German language speaker)
- Expected lack of compliance

8 CRITERIA FOR PATIENT WITHDRAWAL AND STUDY TERMINATION

8.1 Patient withdrawal

Patients are free to withdraw from the study at any time without providing any reason. In general, patients may withdraw from the trial for the following reasons:

- At their own request
- In the investigator's opinion, if the continuation of the trial is detrimental to the patient's well-being

The reason for patient withdrawal will be documented in the (electronic) Case Report Form (eCRF) and in the patient's medical records.

8.2 Study termination

Premature termination of the study is at the discretion of the principal investigator and may occur in any of the following events:

- Medical or ethical reasons affecting the continuation of the study;
- Insufficient patient recruitment.
- External evidence demanding a termination of the trial.

The independent ethics committee has to be informed about termination of the trial

9 METHODS AGAINST BIAS

9.1 Selection bias / Randomization

Consecutive patients are screened and enrolled, if eligible. Group allocation will be performed via a database randomization tool (REDCap) using block randomization stratified by the difficulty of liver resection and type of malignancy which are deemed to be the most important confounders. Subjects withdrawn from the trial retain their identification codes (e.g. ID number). New subjects receive a new identification code.

Randomization is carried out before the study intervention at visit 1. The details of randomization will be kept safe and confidential.

9.2 Performance bias

Treatment of patients follows the German S3-guidelines and the standardized clinical pathways of the Department of Surgery, University Medical Center Mannheim. We expect random occurrence of primary and secondary liver malignancies during recruitment, and we expect random occurrence of variable difficult cases.

9.3 Detection bias

Blinding of patients and study stuff is not feasible due to the technical differences between the study intervention.

9.4 Reporting and publication bias

The trial will be registered in a registry fulfilling ICMJE criteria. The results will be published in an international journal according to the CONSORT Statement.

10 TRIAL IMPLEMENTATION

Patients scheduled for curative-intent minimally invasive (robotic-assisted or laparoscopic) liver surgery will be screened for inclusion into the Roc'n'Roll-Trial and documented in the screening log. Patients meeting the eligibility criteria are asked for participation in the trial. Written informed consent has to be obtained at least on the day prior surgery. Study visits are displayed in the table below.

	**Screening**	**Surgery**	**Follow-Up**			
**Visit**	**1**	**2**	**3**	**4**	**5**	**6**
**Time points, days (surgery = day 0)**	**up to - 30**	**0**	**Day of discharge**	**30**	**60**	**90**
Eligibility criteria<br>Written informed consent	•					
Difficulty score	•					
Baseline demographics	•					
Randomisation		•				
Trial intervention		•				
Assessment of secondary outcome measures			•	•	•	•
Assessment of safety		•	•	•	•	•
Assessment of primary outcome / Quality of life	•			•#	•#	•#*

optional evaluation by electronic mail (email) or telephone, *primary endpoint

Difficulty score	Tumor location, extent of liver resection, tumor size, proximity to major vessels, liver function, hybrid technique
Baseline demographics	Age, sex, ASA-class, BMI, cancer type, relationship status*, employment status, education status, smoking/drinking habits, past medical/surgical history, updated Charlson comorbidity index[33], neoadjuvant therapy, mobility scale
Assessment of secondary outcome measures and safety	Type and extent of liver resection, operation time, total blood loss, conversion rate to open surgery, time-to-functional recovery, postoperative hospital stay, postoperative intensive/intermediate care unit stay, readmission to intermediate/intensive care unit, readmission rate to hospital, invasive interventions, postoperative medical complications, postoperative surgical complications, perioperative morbidity (Clavien-Dindo), pathological details, days in home environment, discharge destination from hospital, return to oncologic intended therapy, HRQoL, Costs within 90 days
Quality of life	QLQ-C30 questionnaire, EQ-5D-5L

*married/living with partner, widowed, divorced, never married

11 ASSESSMENT OF SAFETY

A set of predefined complications will be considered as clinical events and documented in the Roc’n’Roll-Trial (see section 2.3):

- Need for invasive interventions
- Cardiovascular complications (Myocardial infarction, Stroke, Deep venous thrombosis)
- Pulmonary complications (Pneumonia, Atelectasis, Acute Respiratory Distress Syndrome, Pulmonary embolism)
- Postoperative delirium
- Acute kidney insufficiency
- Surgical site infection
- Intraabdominal fluid collection/abscess
- Posthepatectomy complications (bile leakage, liver failure, hemorrhage)

These complications will be graded by the Clavien-Dindo-Classification until 90 days (Appendix D) after surgery.[34] There will be no serious advers event reporting within the Roc’n’Roll trial.

12 STATISTICAL CONSIDERATIONS

12.1 Sample size calculation and statistical analyses

The sample size calculation is based on the primary endpoint the mean quality of life within 90 days after surgery measured by the role functioning scale of the European Organisation for Research and Treatment of Cancer (EORTC) QLQ-C30 questionnaire. A longitudinal analysis of covariance will be performed by estimating a linear mixed model with fixed effects for baseline values, time and interaction between time and group variable. Previously published studies revealed changes up to 20 points in the role functioning scale within 3 months of patients who underwent liver resections for liver maligncancies.[5,9-11,35] In a previous randomized trial a mean difference of 13% in the role functioning scale was determined to be a clinically significant change in patients treated by laparoscopic or open liver surgery.[5] This translates into a "moderate" change of the quality of life.[36] Thus, we hypothetized a mean difference of 13 points (20% standard deviation) in the role functioning scale in favor of robotic-assisted surgery to be clinically relevant. In accordance with the above criteria, a total of 38 patients per group will enable the detection of clinically significant difference (13 points) in the role functioning within 90 days (measured at three time points: 30 days, 60 days, 90 days) at the significance level $\alpha = 0.05$ and a power of 80 % ($\beta = 0.20$). Assuming a conservative attrition rate of 15% including dropouts, withdrawals, or loss to follow-up, a total of 45 patients will provide adequate power for the statistical analysis. Sample size was calculated using SAS version 9.4. The confirmatory analysis is performed based on an intention-to-treat (ITT) population and with respect to ITT principles. The null-hypothesis is that there is no difference between the study groups in the primary variable "role functioning". The aim of the study is to reject this hypothesis and show differences between the groups. The analysis of covariance (ANCOVA), adjusting for age and EORTC QLQ-C30 role functioning score before surgery will be used for the primary endpoint. The average value of HRQoL measures will be calculated and used for primary analysis if HRQoL was observed for at least one postoperative timepoint. If there are no further observations available, missing values for HRQoL measures will be using multiple imputation methods as described previously.[37] A clinically meaningful difference in other QLQ-C30 items was defined as an adjusted change of >10 points from baseline on follow-up between treatment arms, while adjusted score differences of >0.1 and >7 points were used for the EQ-5D index and EQ-5D VAS, respectively.[36,38] Continuous variables will be summarized by sample size, mean, median, standard deviation, minimum, maximum, and interquartile range. Discrete variables will be summarized by frequencies and percentages. The analysis of secondary endpoints will be analogous to that of the primary endpoint. All outcome variables will be analyzed using parametric and non-parametric methods according to scaling and distribution. All statistical tests will be evaluated at the 0.05 - level of significance. Summary statistics for baseline and demographic variables will be provided.

13 DATA MANAGEMENT

13.1 Data collection

The REDCap system will be used to create an electronic case report form (CRF). The CRF will be printed prior to paper-based data collection. The data transmission is encrypted with secure socket layer (SSL) technology and protected by firewalls at the University of Heidelberg. Patients will fill questionnaires for quality of life either on paper-based questionnaire or electronically in the REDCap platform under guidance of staff of the study team with a paper-based source data. If the patient is willing to respond the survey on follow-up by email, the survey will be sent directly from the REDCap survey platform and a paper-based source data. The patients might respond the survey on follow-up by telephone under guidance of staff of the study team. The investigator or a designated representative must enter all protocol-required information in the CRF. The CRF should be completed as soon as possible after the information is collected, preferably on the same day when a trial subject is seen for an examination, treatment, or any other procedure. The reason for missing data should be provided. Changes to data on the REDCap system and paper based CRF. are logged with a timestamp. The completed CRF must be reviewed and signed by the investigator named in the trial protocol or by a designated sub-investigator. The PIs will retain the originals of all CRF at the end of the trial. Data evaluation takes place at the clinical trial center of the Department of Surgery.

13.2 Quality control (Monitoring)

Monitoring within the Roc’n’Roll-Trial is carried out by an independent investigator at the Department of Surgery who is not involved in the trial and in completing the CRFs. The basic data of all participating patients are completely checked, i.e. existing patient, patient number, the availability of signed informed consent and randomization sheet. For a proportion of 10% of the study participants (randomly selected) a complete check of all data in the CRF (i.e. a 100% clinical source data verification; SDV) is carried out. The extent of further SDV is dependent on the quality of the data and the occurrence of protocol violations.

14 ETHICAL CONSIDERATIONS

14.1 Patient protection

The responsible investigator will ensure that this study is conducted in agreement with either the Declaration of Helsinki (Tokyo, Venice, Hong Kong, Somerset West, Edinburgh, Seoul, and Fortaleza amendments) or the laws and regulations of the country, whichever provides the greatest protection of the patient. The protocol has been written, and the study will be conducted according to the ICH Harmonized Tripartite Guideline for Good Clinical Practice. The protocol is approved by the local independent ethics committee.
This trial is funded by the Department of Surgery, University Medical Center Mannheim. There is no external funding for this trial. As all procedures within this trial are part of the clinical routine, all study interventions are covered by the standard hospital liability insurance.

14.2 Informed consent

All patients will be informed of the aims of the study, the possible clinical events/hazards to which he/she will be exposed, and the mechanism of treatment allocation as well as the additional time burden, he/she will be exposed. They will be informed as to the strict confidentiality of their patient data, but that their medical records may be reviewed for trial purposes by authorized individuals other than their treating physician. The patient's consent must be confirmed at the time of consent by the patient’s personally dated signature. The signed consent document is kept by the investigator. A copy of the signed consent document is handed out to the subject.
It will be emphasized that participation is voluntary and that the patient is allowed to refuse further participation in the protocol whenever he/she wants. This will not prejudice the patient’s subsequent care. Documented informed consent must be obtained for all patients included in the study before they are registered in the study. This must be done in accordance with the national and local regulatory requirements.

15 REGULATORY OBLIGATIONS

15.1 Independent Ethics Committee (IEC)

Prior to the start of the trial the protocol, the informed consent form, and other written subject information must be submitted to the IEC for written approval. Formal approval by the IEC should preferably mention the title of the trial, the trial code, the trial site, and any other documents reviewed. It must mention the date on which the decision was made and must be officially signed by a committee member. Following IEC approval all subsequent protocol amendments and changes to the informed consent document must be approved by the IEC. The EC must be informed of the end of the trial. The investigator must keep a record of all communications with the IEC and the regulatory authorities.

15.2 Patient confidentiality

It is the responsibility of the investigator to maintain the patient´s confidentiality. During the trial, patients will be identified solely by means of their year of birth and individual identification code (screening number, randomization number). Trial findings will be stored in accordance with local data protection law/ICH GCP (International Conference on Harmonization of Technical Requirements for Registration of Pharmaceuticals for Human Use / Good Clinical Practice) Guidelines and will be handled in strictest confidence. For the protection of these data, organizational procedures are implemented to prevent the distribution of data to unauthorized people. In accordance with local data protection law/ICH GCP Guidelines, it is required that the investigator and institution must permit authorized representatives (e.g. monitor(s), regulatory agency(s), and the IEC) to inspect the patient-related data collected during the trial. The investigator will maintain a personal subject identification list to enable records to be identified.

REFERENCES

1. Ito H, Are C, Gonen M, et al. Effect of postoperative morbidity on long-term survival after hepatic resection for metastatic colorectal cancer. Ann Surg 2008;247(6):994-1002. DOI: 10.1097/SLA.0b013e31816c405f.
2. Chauhan A, House MG, Pitt HA, et al. Post-operative morbidity results in decreased long-term survival after resection for hilar cholangiocarcinoma. HPB (Oxford) 2011;13(2):139-47. DOI: 10.1111/j.1477-2574.2010.00262.x.
3. Chok KS, Chan MM, Dai WC, et al. Survival outcomes of hepatocellular carcinoma resection with postoperative complications - a propensity-score-matched analysis. Medicine (Baltimore) 2017;96(12):e6430. DOI: 10.1097/MD.0000000000006430.
4. Chie WC, Yu F, Li M, et al. Quality of life changes in patients undergoing treatment for hepatocellular carcinoma. Qual Life Res 2015;24(10):2499-506. DOI: 10.1007/s11136-015-0985-8.
5. Fretland AA, Dagenborg VJ, Waaler Bjornelv GM, et al. Quality of life from a randomized trial of laparoscopic or open liver resection for colorectal liver metastases. Br J Surg 2019;106(10):1372-1380. DOI: 10.1002/bjs.11227.
6. Gamper EM, Petersen MA, Aaronson N, et al. Development of an item bank for the EORTC Role Functioning Computer Adaptive Test (EORTC RF-CAT). Health Qual Life Outcomes 2016;14:72. DOI: 10.1186/s12955-016-0475-x.
7. Meier A, Yopp A, Mok H, Kandunoori P, Tiro J, Singal AG. Role functioning is associated with survival in patients with hepatocellular carcinoma. Qual Life Res 2015;24(7):1669-75. DOI: 10.1007/s11136-014-0895-1.
8. Andersen LW, Kim WY, Chase M, et al. The prevalence and significance of abnormal vital signs prior to in-hospital cardiac arrest. Resuscitation 2016;98:112-7. DOI: 10.1016/j.resuscitation.2015.08.016.
9. Studer P, Horn T, Haynes A, Candinas D, Banz VM. Quality of life after hepatic resection. Br J Surg 2018;105(3):237-243. DOI: 10.1002/bjs.10735.
10. Rees JR, Blazeby JM, Fayers P, et al. Patient-reported outcomes after hepatic resection of colorectal cancer metastases. J Clin Oncol 2012;30(12):1364-70. DOI: 10.1200/JCO.2011.38.6177.
11. Miller AR, St Hill CR, Ellis SF, Martin RC. Health-related quality of life changes following major and minor hepatic resection: the impact of complications and postoperative anemia. Am J Surg 2013;206(4):443-50. DOI: 10.1016/j.amjsurg.2013.02.011.
12. Abu Hilal M, Aldrighetti L, Dagher I, et al. The Southampton Consensus Guidelines for Laparoscopic Liver Surgery: From Indication to Implementation. Ann Surg 2018;268(1):11-18. DOI: 10.1097/SLA.0000000000002524.
13. Fretland AA, Dagenborg VJ, Bjornelv GMW, et al. Laparoscopic Versus Open Resection for Colorectal Liver Metastases: The OSLO-COMET Randomized Controlled Trial. Ann Surg 2018;267(2):199-207. DOI: 10.1097/SLA.0000000000002353.
14. Idrees K, Bartlett DL. Robotic liver surgery. Surg Clin North Am 2010;90(4):761-74. DOI: 10.1016/j.suc.2010.04.020.
15. Birgin E, Kaslow SR, Hetjens S, Correa-Gallego C, Rahbari NN. Minimally Invasive versus Open Liver Resection for Stage I/II Hepatocellular Carcinoma. Cancers (Basel) 2021;13(19). DOI: 10.3390/cancers13194800.
16. Guan R, Chen Y, Yang K, et al. Clinical efficacy of robot-assisted versus laparoscopic liver resection: a meta analysis. Asian J Surg 2019;42(1):19-31. DOI: 10.1016/j.asjsur.2018.05.008.
17. Tsung A, Geller DA, Sukato DC, et al. Robotic versus laparoscopic hepatectomy: a matched comparison. Ann Surg 2014;259(3):549-55. DOI: 10.1097/SLA.0000000000000250.
18. Kamarajah SK, Bundred J, Manas D, Jiao LR, Hilal MA, White SA. Robotic Versus Conventional Laparoscopic Liver Resections: A Systematic Review and Meta-Analysis. Scand J Surg 2020:1457496920925637. DOI: 10.1177/1457496920925637.
19. Ciria R, Berardi G, Alconchel F, et al. The impact of robotics in liver surgery: A worldwide systematic review and short-term outcomes meta-analysis on 2,728 cases. J Hepatobiliary Pancreat Sci 2020. DOI: 10.1002/jhbp.869.
20. Aaronson NK, Ahmedzai S, Bergman B, et al. The European Organization for Research and Treatment of Cancer QLQ-C30: a quality-of-life instrument for use in international clinical trials in oncology. J Natl Cancer Inst 1993;85(5):365-76. DOI: 10.1093/jnci/85.5.365.
21. Wakabayashi G. What has changed after the Morioka consensus conference 2014 on laparoscopic liver resection? Hepatobiliary Surg Nutr 2016;5(4):281-9. DOI: 10.21037/hbsn.2016.03.03.
22. Wong-Lun-Hing EM, van Dam RM, van Breukelen GJ, et al. Randomized clinical trial of open versus laparoscopic left lateral hepatic sectionectomy within an enhanced recovery after surgery programme (ORANGE II study). Br J Surg 2017;104(5):525-535. DOI: 10.1002/bjs.10438.

23.Cassidy MR, Rosenkranz P, McCabe K, Rosen JE, McAneny D. I COUGH: reducing postoperative pulmonary complications with a multidisciplinary patient care program. JAMA Surg 2013;148(8):740-5. DOI: 10.1001/jamasurg.2013.358.
24.Canet J, Gallart L, Gomar C, et al. Prediction of postoperative pulmonary complications in a population-based surgical cohort. Anesthesiology 2010;113(6):1338-50. DOI: 10.1097/ALN.0b013e3181fc6e0a.
25.Force ADT, Ranieri VM, Rubenfeld GD, et al. Acute respiratory distress syndrome: the Berlin Definition. JAMA 2012;307(23):2526-33. DOI: 10.1001/jama.2012.5669.
26.Humeidan ML, Otey A, Zuleta-Alarcon A, Mavarez-Martinez A, Stoicea N, Bergese S. Perioperative Cognitive Protection-Cognitive Exercise and Cognitive Reserve (The Neurobics Trial): A Single-blind Randomized Trial. Clin Ther 2015;37(12):2641-50. DOI: 10.1016/j.clinthera.2015.10.013.
27.Myles PS, Bellomo R, Corcoran T, et al. Restrictive versus Liberal Fluid Therapy for Major Abdominal Surgery. N Engl J Med 2018;378(24):2263-2274. DOI: 10.1056/NEJMoa1801601.
28.Rahbari NN, Garden OJ, Padbury R, et al. Posthepatectomy liver failure: A definition and grading by the International Study Group of Liver Surgery (ISGLS). Surgery 2011 (In Eng). DOI: 10.1016/j.surg.2010.10.001.
29.de la Plaza Llamas R, Ramia Angel JM, Bellon JM, et al. Clinical Validation of the Comprehensive Complication Index as a Measure of Postoperative Morbidity at a Surgical Department: A Prospective Study. Ann Surg 2018;268(5):838-844. DOI: 10.1097/SLA.0000000000002839.
30.Bell M, Eriksson LI, Svensson T, et al. Days at Home after Surgery: An Integrated and Efficient Outcome Measure for Clinical Trials and Quality Assurance. EClinicalMedicine 2019;11:18-26. DOI: 10.1016/j.eclinm.2019.04.011.
31.van Hilst J, Strating EA, de Rooij T, et al. Costs and quality of life in a randomized trial comparing minimally invasive and open distal pancreatectomy (LEOPARD trial). Br J Surg 2019;106(7):910-921. DOI: 10.1002/bjs.11147.
32.Grochtdreis T, Dams J, Konig HH, Konnopka A. Health-related quality of life measured with the EQ-5D-5L: estimation of normative index values based on a representative German population sample and value set. Eur J Health Econ 2019;20(6):933-944. DOI: 10.1007/s10198-019-01054-1.
33.Quan H, Li B, Couris CM, et al. Updating and validating the Charlson comorbidity index and score for risk adjustment in hospital discharge abstracts using data from 6 countries. Am J Epidemiol 2011;173(6):676-82. DOI: 10.1093/aje/kwq433.
34.Slankamenac K, Graf R, Barkun J, Puhan MA, Clavien PA. The comprehensive complication index: a novel continuous scale to measure surgical morbidity. Ann Surg 2013;258(1):1-7. DOI: 10.1097/SLA.0b013e318296c732.
35.Heise D, Bayings W, Tuinhof A, et al. Long-term outcome and quality of life after initial and repeat resection of colorectal liver metastasis: A retrospective analysis. Int J Surg 2017;48:281-285. DOI: 10.1016/j.ijsu.2017.11.032.
36.Osoba D, Rodrigues G, Myles J, Zee B, Pater J. Interpreting the significance of changes in health-related quality-of-life scores. J Clin Oncol 1998;16(1):139-44. (In eng). DOI: 10.1200/jco.1998.16.1.139.
37.Diener MK, Huttner FJ, Kieser M, et al. Partial pancreatoduodenectomy versus duodenum-preserving pancreatic head resection in chronic pancreatitis: the multicentre, randomised, controlled, double-blind ChroPac trial. Lancet 2017;390(10099):1027-1037. DOI: 10.1016/S0140-6736(17)31960-8.
38.Pickard AS, Neary MP, Cella D. Estimation of minimally important differences in EQ-5D utility and VAS scores in cancer. Health Qual Life Outcomes 2007;5:70. (In eng). DOI: 10.1186/1477-7525-5-70.

SIGNATURES

Name: PD Dr. Emrullah Birgin, MD
Position: Principal Investigator

Date: ____________ Signature: ________________________________

Name: Prof. Dr. Nuh N. Rahbari, MD
Position: Principal Investigator

Date: ____________ Signature: ________________________________

Name: Dr. sc. Svetlana Hetjens
Position: Biostatistician

Date: ____________ Signature: ________________________________

TRIAL FLOW CHART

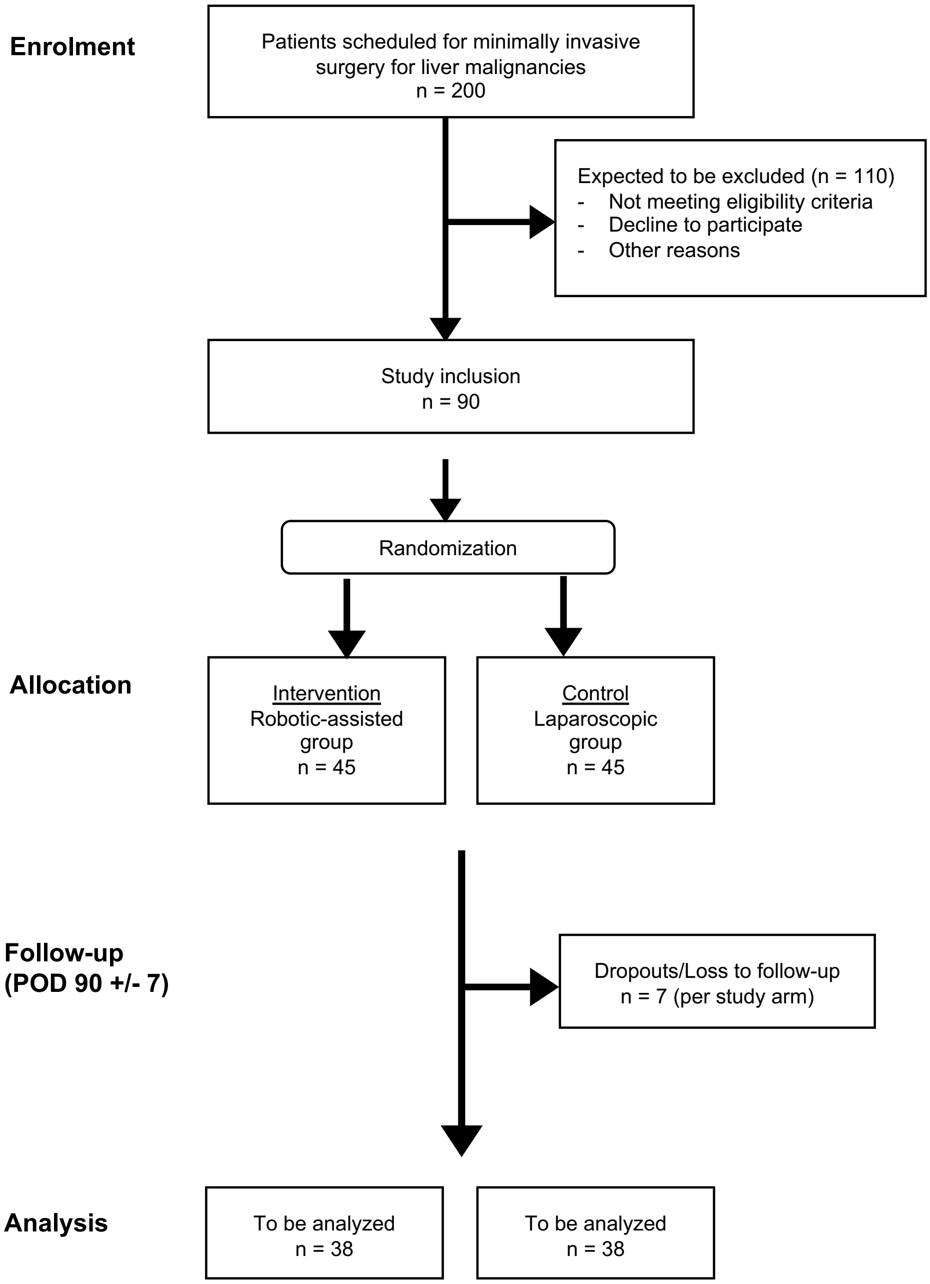

APPENDIX A: QLQ-C30 QUESTIONNAIRE

ENGLISH

EORTC QLQ-C30 (version 3)

We are interested in some things about you and your health. Please answer all of the questions yourself by circling the number that best applies to you. There are no "right" or "wrong" answers. The information that you provide will remain strictly confidential.

Please fill in your initials: |_|_|_|_|
Your birthdate (Day, Month, Year): |_|_|_|_|_|_|_|_|
Today's date (Day, Month, Year): 31 |_|_|_|_|_|_|_|_|

SPECIMEN

	Not at All	A Little	Quite a Bit	Very Much
1. Do you have any trouble doing strenuous activities, like carrying a heavy shopping bag or a suitcase?	1	2	3	4
2. Do you have any trouble taking a <u>long</u> walk?	1	2	3	4
3. Do you have any trouble taking a <u>short</u> walk outside of the house?	1	2	3	4
4. Do you need to stay in bed or a chair during the day?	1	2	3	4
5. Do you need help with eating, dressing, washing yourself or using the toilet?	1	2	3	4

**During the past week:**	Not at All	A Little	Quite a Bit	Very Much
6. Were you limited in doing either your work or other daily activities?	1	2	3	4
7. Were you limited in pursuing your hobbies or other leisure time activities?	1	2	3	4
8. Were you short of breath?	1	2	3	4
9. Have you had pain?	1	2	3	4
10. Did you need to rest?	1	2	3	4
11. Have you had trouble sleeping?	1	2	3	4
12. Have you felt weak?	1	2	3	4
13. Have you lacked appetite?	1	2	3	4
14. Have you felt nauseated?	1	2	3	4
15. Have you vomited?	1	2	3	4
16. Have you been constipated?	1	2	3	4

Please go on to the next page

ENGLISH

During the past week:	Not at All	A Little	Quite a Bit	Very Much
17. Have you had diarrhea?	1	2	3	4
18. Were you tired?	1	2	3	4
19. Did pain interfere with your daily activities?	1	2	3	4
20. Have you had difficulty in concentrating on things, like reading a newspaper or watching television?	1	2	3	4
21. Did you feel tense?	1	2	3	4
22. Did you worry?	1	2	3	4
23. Did you feel irritable?	1	2	3	4
24. Did you feel depressed?	1	2	3	4
25. Have you had difficulty remembering things?	1	2	3	4
26. Has your physical condition or medical treatment interfered with your family life?	1	2	3	4
27. Has your physical condition or medical treatment interfered with your social activities?	1	2	3	4
28. Has your physical condition or medical treatment caused you financial difficulties?	1	2	3	4

For the following questions please circle the number between 1 and 7 that best applies to you

29. How would you rate your overall health during the past week?

1	2	3	4	5	6	7
Very poor						Excellent

30. How would you rate your overall quality of life during the past week?

1	2	3	4	5	6	7
Very poor						Excellent

SPECIMEN

© Copyright 1995 EORTC Quality of Life Group. All rights reserved. Version 3.0

APPENDIX B: DIFFICULTY SCORING SYSTEM

IWATE Criteria

Difficulty index	0	1	2	3	4	5	6	7	8	9	10	11	12
Difficulty level	Low				Intermediate			Advanced			Expert		

Index surgery: Simple and small partial hepatectomy in segment III; Left lateral sectionectomy; Right or left hepatectomy; Posterior sectionectomy for segment VII tumor ≥ 3 cm

Scoring system

Tumor location (Couinaud segment)

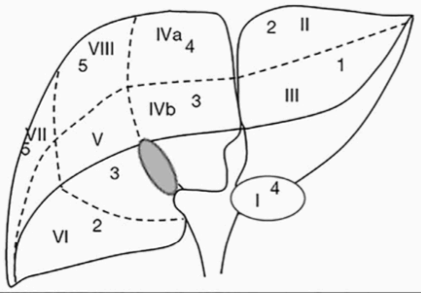

Segment	Score
S1	4
S2	2
S3	1
S4a	4
S4b	3
S5	3
S6	2
S7	5
S8	5

Tumor size

	Score
<3 cm	0
≥3 cm	1

Proximity to major vessel*

	Score
No	0
Yes	1

*Main or second branch of Glisson's tree, major hepatic vein, or inferior vena cava

Extent of liver resection

	Score
Partial resection	0
Left lateral sectionectomy	2
Segmentectomy	3
Sectionectomy and more	4

HALS/Hybrid

	Score
No	0
Yes	−1

Liver function

	Score
Child Pugh A	0
Child Pugh B	1

APPENDIX B: EQ-5D-5L

Under each heading, please tick the ONE box that best describes your health TODAY.

MOBILITY

I have no problems in walking about ❑
I have slight problems in walking about ❑
I have moderate problems in walking about ❑
I have severe problems in walking about ❑
I am unable to walk about ❑

SELF-CARE

I have no problems washing or dressing myself ❑
I have slight problems washing or dressing myself ❑
I have moderate problems washing or dressing myself ❑
I have severe problems washing or dressing myself ❑
I am unable to wash or dress myself ❑

USUAL ACTIVITIES *(e.g. work, study, housework, family or leisure activities)*

I have no problems doing my usual activities ❑
I have slight problems doing my usual activities ❑
I have moderate problems doing my usual activities ❑
I have severe problems doing my usual activities ❑
I am unable to do my usual activities ❑

PAIN / DISCOMFORT

I have no pain or discomfort ❑
I have slight pain or discomfort ❑
I have moderate pain or discomfort ❑
I have severe pain or discomfort ❑
I have extreme pain or discomfort ❑

ANXIETY / DEPRESSION

I am not anxious or depressed ❑
I am slightly anxious or depressed ❑
I am moderately anxious or depressed ❑
I am severely anxious or depressed ❑
I am extremely anxious or depressed ❑

Sample

- We would like to know how good or bad your health is TODAY.
- This scale is numbered from 0 to 100.
- 100 means the best health you can imagine.
  0 means the worst health you can imagine.
- Please mark an X on the scale to indicate how your health is TODAY.
- Now, write the number you marked on the scale in the box below.

YOUR HEALTH TODAY = ☐

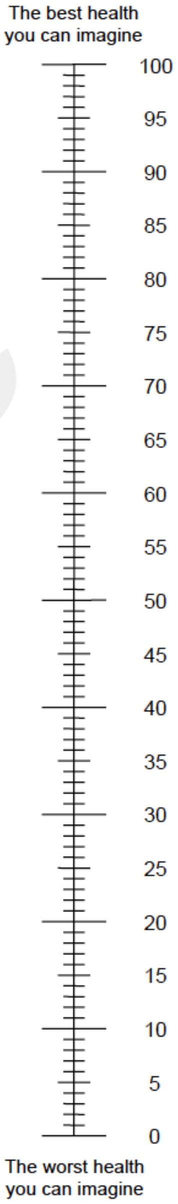

Sample

APPENDIX D: CLASSIFICATION OF SURGICAL COMPLICATIONS

TABLE 1. Classification of Surgical Complications

Grade	Definition
Grade I	Any deviation from the normal postoperative course without the need for pharmacological treatment or surgical, endoscopic, and radiological interventions
	Allowed therapeutic regimens are: drugs as antiemetics, antipyretics, analgetics, diuretics, electrolytes, and physiotherapy. This grade also includes wound infections opened at the bedside
Grade II	Requiring pharmacological treatment with drugs other than such allowed for grade I complications
	Blood transfusions and total parenteral nutrition are also included
Grade III	Requiring surgical, endoscopic or radiological intervention
Grade IIIa	Intervention not under general anesthesia
Grade IIIb	Intervention under general anesthesia
Grade IV	Life-threatening complication (including CNS complications)* requiring IC/ICU management
Grade IVa	Single organ dysfunction (including dialysis)
Grade IVb	Multiorgan dysfunction
Grade V	Death of a patient
Suffix “d”	If the patient suffers from a complication at the time of discharge (see examples in Table 2), the suffix “d” (for “disability”) is added to the respective grade of complication. This label indicates the need for a follow-up to fully evaluate the complication.

*Brain hemorrhage, ischemic stroke, subarrachnoidal bleeding, but excluding transient ischemic attacks.
CNS, central nervous system; IC, intermediate care; ICU, intensive care unit.

APPENDIX E: MOBILITY SCALE

	Items	Response categories
1.	Can you, fully independently, get in and out of bed?	Yes / No
2.	Can you, fully independently, stand up from sitting in a chair?	Yes / No
3.	Can you, fully independently, walk around in your room (if necessary with a crutch or rollator)?	Yes / No
4.	Can you, fully independently, get around in the ward (if necessary with a crutch or rollator)?	Yes / No
5.	Can you, fully independently, go up and down stairs?	Yes / No
6.	Can you, fully independently, get on and off the toilet?	Yes / No
7.	Can you, fully independently, wash your face and hands?	Yes / No
8.	Can you, fully independently, wash and dry your whole body?	Yes / No
9.	Can you, fully independently, dress yourself?	Yes / No
10.	Can you, fully independently, feed yourself?	Yes / No

When the patient is able to perform 8 of the 10 items, patient will be considered to be independently mobile. Patients will also be considered to fulfill the discharge criteria regarding independent mobility, if they reach the preoperative level.
